# A survey on extremism analysis using natural language processing: definitions, literature review, trends and challenges

**DOI:** 10.1007/s12652-021-03658-z

**Published:** 2022-01-12

**Authors:** Javier Torregrosa, Gema Bello-Orgaz, Eugenio Martínez-Cámara, Javier Del Ser, David Camacho

**Affiliations:** 1 Computer Systems Engineering Department, Universidad Politécnica de Madrid, Madrid, Spain; 2 Department of Computer Science and Artificial Intelligence, Andalusian Research Institute in Data Science and Computational Intelligence (DaSCI), University of Granada, Granada, Spain; 3 TECNALIA, Basque Research and Technology Alliance (BRTA), Mendaro, Spain

**Keywords:** Natural language processing, Radicalization, Extremism, Machine learning, Deep learning

## Abstract

Extremism has grown as a global problem for society in recent years, especially after the apparition of movements such as jihadism. This and other extremist groups have taken advantage of different approaches, such as the use of Social Media, to spread their ideology, promote their acts and recruit followers. The extremist discourse, therefore, is reflected on the language used by these groups. Natural language processing (NLP) provides a way of detecting this type of content, and several authors make use of it to describe and discriminate the discourse held by these groups, with the final objective of detecting and preventing its spread. Following this approach, this survey aims to review the contributions of NLP to the field of extremism research, providing the reader with a comprehensive picture of the state of the art of this research area. The content includes a first conceptualization of the term extremism, the elements that compose an extremist discourse and the differences with other terms. After that, a review description and comparison of the frequently used NLP techniques is presented, including how they were applied, the insights they provided, the most frequently used NLP software tools, descriptive and classification applications, and the availability of datasets and data sources for research. Finally, research questions are approached and answered with highlights from the review, while future trends, challenges and directions derived from these highlights are suggested towards stimulating further research in this exciting research area.

## Introduction

The rise of Social Media platforms has strengthened the interest of researchers for studying human behavior on different contexts, as they give them the chance of crawling real time data from the users, but also stored or published data during long periods of time (Bayerl et al. 2014). Since most of the content published on the Internet is in text format, it is unsurprising that one of the most frequently used approaches for online pattern extraction comes from natural language processing (NLP). This artificial intelligence area uses a set of computational methods for making human language accessible to computers, and more specifically for giving the computers the ability to understand and generate human language (Eisenstein 2019; Indurkhya and Damerau 2010). NLP techniques are used in both academia and industry for text analysis applications, such as medicine (Wang et al. 2018; Savova et al. 2019; Tiwari et al. 2020), mental health (Calvo et al. 2017; Stewart and Velupillai 2021), economy (Fisher et al. 2016) or crime prevention (Schmidt and Wiegand 2017).

One of the area that has benefited of NLP techniques on recent years is the study of extremist discourse, particularly due to the increasing use of Social Media by different extremist groups. Social Media platforms, such as Twitter or Facebook, have changed the way extremists communicate, recruit and disseminate their ideas (Dean et al. 2012). The rise of groups such as Islamic State or the Alt-right, together with their use of these platforms with different objectives (Jawhar 2016; Aliapoulios et al. 2021), has represented a threat for many countries, specially considering that extremism can facilitate the justification of violent actions to achieve a movement’s agenda (Thomas 2012). This threat led different countries to finance research projects and other initiatives related to the study of the traces that extremists users left online, with the aim of identifying early behaviors to stop them before embracing violent extremism. In fact, during the worst days of the jihadist threat (between 2015 and 2018), the European Union invested in several research projects grounded in NLP to track terrorism and online extremism (Bouzar 2018; Fernandez and Alani 2018; Florea et al. 2019; Torregrosa and Panizo 2018). The core of most of the initiatives aim to counter this phenomenon, detecting and classifying extremist content that could lead people to adopt these ideologies. Machine learning (ML) techniques made a great contribution to this purpose (see, for example Scanlon and Gerber 2014).

After the fruitful period of research from different perspectives aimed to study and analyze the extremism phenomenon, a few systematic surveys have approached the specific relationship between NLP and extremism research. These systematic reviews can be divided in two types. The first type has analyzed NLP contributions to areas conceptually related to extremism, such as hate speech (Fortuna and Nunes 2018) or law enforcement (Edwards et al. 2015). The second type gravitates on extremism, including NLP as a key part of its identification (Aldera et al. 2021; Gaikwad et al. 2021).

The limitations of the first approach is quite obvious, as the phenomenon is not studied directly. For example, while studying hate speech has a direct impact on the knowledge about extremism (as hate speech is used by extremists), the latter is a more complex phenomenon, composed by other discursive characteristics. The reviews under the second approach, while do approach the issue directly, actually present two limitations. On one hand, their content is restricted to the specific task of detection, not covering the rest of the whole data mining process (Gaikwad et al. 2021). On the other hand, their lack of depth when studying the NLP approaches under focus (Aldera et al. 2021), missing to provide a thorough description of the diverse spectrum of techniques used in both descriptive and detection processes.

This article aims to cover the gap left by this prior work and other similar surveys through several contributions. First, it helps the audience conceptualize the concept of extremism and extremist discourse, and describes the concepts that can be related or be a core part of these fields. Second, it places an emphasis on NLP contributions to extremism analysis (including both description and classification/detection tasks), with a more comprehensive and critical approach on the different types of NLP techniques used to date. Third, we list several software resources that can be helpful for future research works on this area. Finally, our work discusses on future trends and challenges that shall be confronted in extremism analysis. To this end, a systematic review is conducted to collect and critically analyze the literature regarding NLP applied to the study of extremism. Five research questions are formulated to orchestrate the contributions of this review:

- RQ1. What are the current topics and contributions from NLP to extremism research?
- RQ2. What NLP techniques are used in extremism research?
- RQ3. How have NLP techniques been applied in the field of extremism research?
- RQ4. What NLP software tools are commonly used in extremism research?
- RQ5. Which publicly available datasets/data sources have been used to conduct NLP experiments in extremism research?

**Fig. 1:**
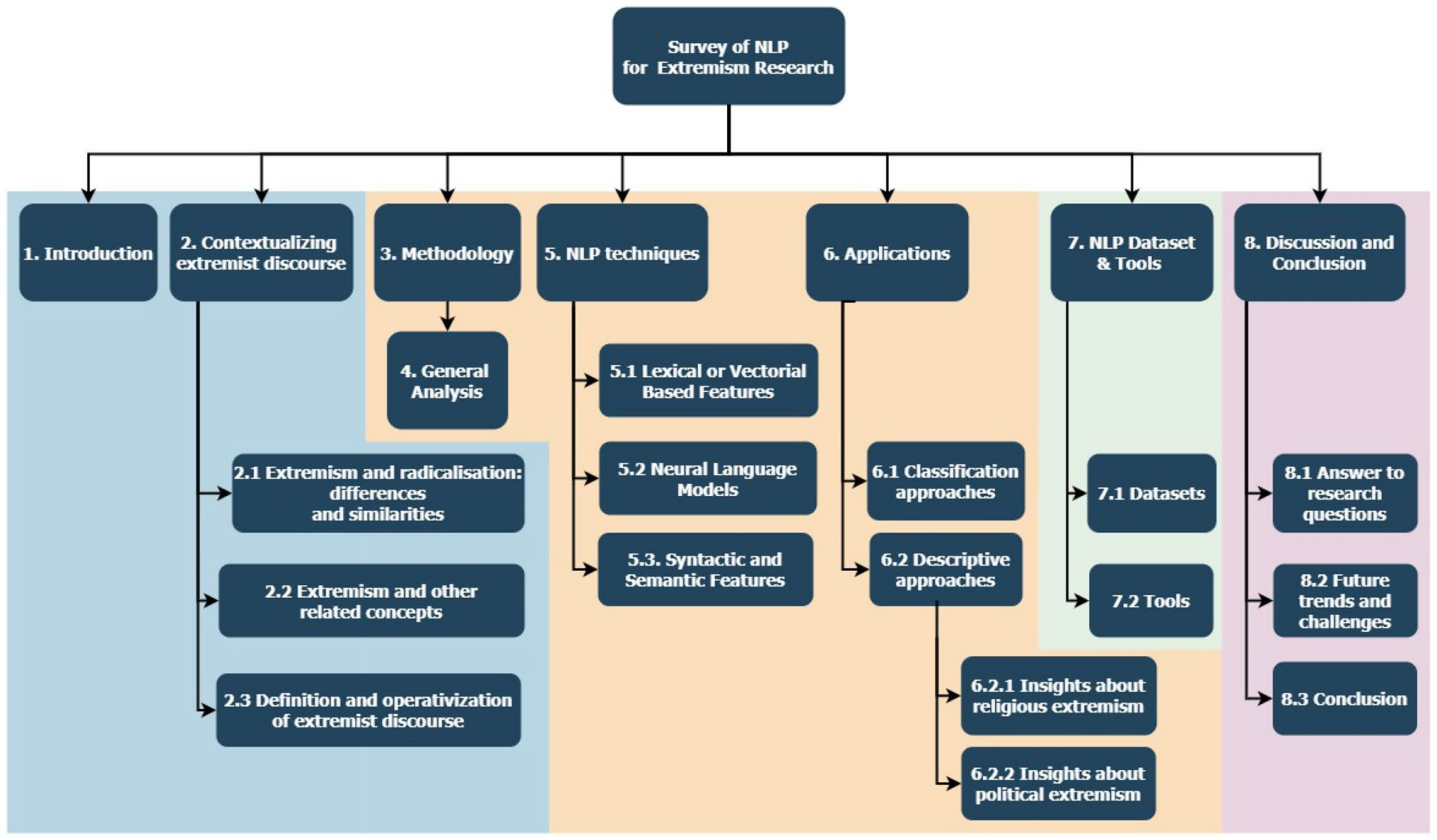
Overall structure of the review. Blue color: theoretical conceptualization; Yellow color: literature analysis; green color: tools; Pink: prospective analysis

Derived from these research questions and the process of answering them, the main contributions of the article can be summarized as follows:

1. It provides a general picture of the theoretical foundations behind the concept of “extremism”, discussing its differences and similarities with other concepts that are often confused or misused as synonyms in the literature.
2. It briefly defines the concept of *extremist discourse*, including some key elements that are present in this type of discourses.
3. It presents an updated picture of the NLP techniques (including preprocessing techniques) used in extremism research, together with an analysis and comparison of their advantages and downsides.
4. It summarizes the different applications that NLP techniques can have on extremism research, such as discourse description and classification. The main ML algorithms used to identify extremist content are also highlighted.
5. It presents different available software tools, together with open datasets and data sources regarding extremism, which can be of utmost help for authors interested in conducting experiments or making advances in this field in the future.
6. It highlights trends, challenges and research directions that can be pursued in this field, supporting them with the conclusions drawn from the analysis.

A summary of the structure of the rest of the overview can be seen in Fig. 1. Section 2 defines the concept of extremism, the differences among extremism and other topics and the distinctive features of the extremist discourse. Section 3 explains how the review was planned and conducted, including the criteria adopted for the inclusion and exclusion of literature, and a brief summary of the process. Section 4 performs a general descriptive analysis of the outcomes of the search conducted, including the trends of publication and the main keywords associated to the articles. Section 5 describes and compares the different NLP techniques used by the authors. Section 6 focuses on the applications of these techniques, dividing them in two approaches: text description and text classification, including the ML algorithms used for this task. Section 7 lists open NLP datasets, data sources and tools used by the authors. Finally, Sect. 8 answers the research questions, presents future trends, challenges and directions of the area, and draws final conclusions.

## Contextualizing the concept of extremist discourse

The definition of extremism has traditionally led to different misconceptions in the literature, specially for authors with low background on social sciences. This section deals with the different definitions around this topic. Section 2.1 analysis the differences between extremism and radicalization, two concepts that are frequently used indistinctly (Schmid 2013). Section 2.2 briefly presents other concepts related to extremism, including definitions and relationships with it. Finally, Sect. 2.3 presents how the concept of extremism will be used in this article, including an operationalization of the extremist language that will act as a framework on which the different articles reviewed can be compared.

### Extremism and radicalization: differences and similarities

The literature so far shows that extremism and radicalization are often used as synonyms or exchangeable terms to refer to the same phenomenon, which engenders the false idea that both terms mean the same. However, while authors do not usually distinguish between them from a methodological perspective, there are indeed theoretical differences that make both terms conceptually different from each other. Actually there is no academic consensus about the definitions of extremism and radicalization (van de Weert and Eijkman 2019). However, the different perspectives concerning their relationship can be summarized in three main approaches:

1. Both concepts are synonyms: This could be related to the use of both terms in the political discourse, which has transformed them into pejorative concepts that are used indistinctly (Schmid 2013).
2. Both concepts are different, but one of them subsumes the other: In this line, several articles use the concept of radicalization as a term to refer to the psychological process previous to the involvement in terrorism and extremism (Schuurman and Taylor 2018).
3. Both concepts are different, without a necessary relationship among them: Regarding this approach, Botticher (Bötticher 2017) conducted a deep analysis of the historical roots of these concepts, in an informed attempt at defining the differences among them. Essentially, the term *radicalization* was born during the 18th century as a way to define a movement against the establishment, but not inherently violent or positioned against democratic values. Meanwhile, the concept of *extremism* refers to an anti-democratic movement, and stands against “all those who do not embrace its dogmatic recipe for a transformation of society”. Another reference to this article can be found in Schuurman and Taylor (Schuurman and Taylor 2018), which highlight that radicalization, understood in its historical context, does not necessarily imply a negative connotation of “change” of the socio-political order, while extremism does.

When it comes to the present review, it is necessary to have an open position towards the three different approaches. Extremism will be considered the core concept of this review, and therefore it will be used as a keyword instead of radicalization, as all the social movements of interest for this article are, essentially, those against democratic values. However, due to the misconception or confusing use of both terms in the literature, both radicalization and extremism will be used as keywords to conduct the search on the databases during the article gathering process. Accordingly, we will include articles from authors considering both terms as synonyms, as well as those using one as part of the other.

### Extremism and other related concepts

**Fig. 2:**
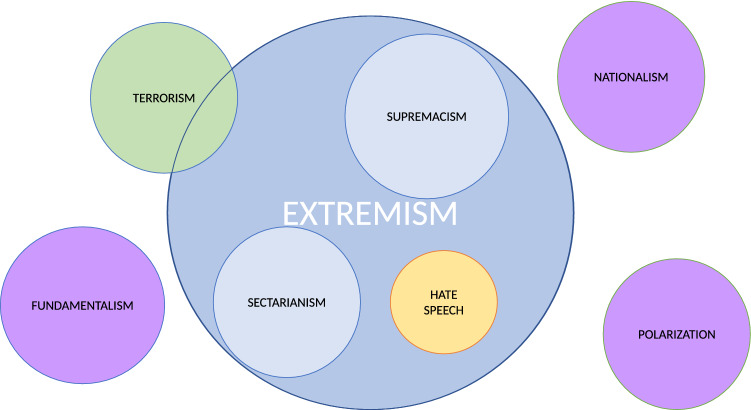
Graphic representation of the overlap between extremism and concepts usually mentioned in the same context. A deeper analysis can be found on Table 1

Similarly to the extremism and radicalization terms, there are other concepts that are currently confusing on their use in the context of extremism research. While some of these terms are quite related, they do not share the same theoretical definition. Figure 2 shows the overlapping between different concepts usually related to extremism, graphically representing different possibilities: actual overlapping (blue), absence of relationship (purple), characteristics of the extremist discourse (yellow) or concepts partially related (green).

Table 1 explains the differences displayed on Fig. 2. This explanation includes the term’s definitions, their difference with the concept of extremism and an example from the literature regarding them. Taking into account that the main characteristic to classify a movement as extremist is that it goes against democratic values, we can find three different types of concepts related to extremism in this table. The first two terms (*supremacism* and *sectarianism*) are actually subtypes of extremism, since they are both different types of ideological movements that aim to suppress or limit certain fundamental democratic values of other social groups. When these ideological movements against democratic values resort to violence to achieve their objectives, it can be said that they constitute a type of *terrorism* (third term in the table). Finally, the last three terms (*polarization*, *fundamentalism* and *nationalism*), although are related to extremism, do not necessarily share its opposition against democratic values.

There are other concepts that, despite apparently related to extremism, are just manifestations of the violence and discrimination underlying this concept. Some examples are hate speech (Olteanu et al. 2018), racism (Fuchs 2016) or stalking/cyber-stalking (Kruglanski et al. 2020). The creation of fake news (Spohr 2017; Bozarth and Budak 2020) and its relationship with extremism currently represents another rising problem that has attracted the attention from the research community.

**Table 1:** Concepts, definitions and distinction from extremism

Concept	Definition	Distinction from extremism	Example of the concept
Supremacism	The ideology that one group is naturally superior to another one, due to their race, sex, economic status, nation, etc. (Schaefer 1990)	Supremacism would be a subtype of extremism, as supremacist groups are contrary to the existence of equal rights	White supremacist movement (Kantrowitz 2015) or chinese supremacism (Leibold 2010)
Sectarianism	Form of discrimination between groups based on a specific factor. For years, it was limited to religion, but nowadays this concept is technically similar to supremacism (Phillips 2015)	As with supremacism, sectarianism would be a sub-type of extremism, as it is contrary to the existence of equal rights	There are several examples of sectarian groups, such as the Islamic State (Roy 2017)
Terrorism	Systematic use of violence, propaganda and fear towards and specific population to achieve ideological objectives (López et al. 2016)	Terrorism always implies violence, while extremism does not necessarily use it. However, both are against one or more fundamental values of a society	There are many examples of terrorism, both in national and international contexts, such as IRA in Ireland/North Ireland (Pruitt 2007), ETA in Spain (Shepard 2002), FARC in Colombia (Saab and Taylor 2009) or Al’Qaeda (Burke 2004)
Polarization	Ideological movement towards a more extreme point of view in whatever direction is indicated by the member’s predeliberation tendency (Sunstein 1999)	As occurs with radicalization, polarization is not necessarely violent or against fundamental values of a society	Political or “partisan” polarization (Prior 2013)
Fundamentalism	Tendency to follow literally certain dogmas or ideologies from the “fundamental” and unchangeable practices of the past. As sectarianism, it has a religious connotation (Hunsberger 1995)	Fundamentalism is not necessarely violent or against democratic values	The “Amish” are an example of christian fundamentalist group (Hill and Williamson 2005), and “Gus emunim” was an example of a jew fundamentalist group (Emerson and Hartman 2006)
Nationalism	Ideology based on the nodal point “nation”, on which a community is tied to a certain space, and that is structured through the opposition between the nation and different outgroups (De Cleen 2017)	Nationalism does not necessarily imply a negative connotation. When it turns extremist, it would convert to supremacism	Catalonia, Scotland and Canada have some renowned political movements related to nationalism (Keating 1996)
Hate speech	Language that incites violence or hate against groups, based on specific characteristics, and that can be used with different linguistic styles, such as humour (Fortuna and Nunes 2018)	While extremist discourses frequently include hate speech, they both target different audiences (general public vs minorities) and show different objectives (activation vs discrimination). Also, extremism discourse includes more topics than hate speech, such as recruitment or persuasion (McNamee et al. 2010; Gelber 2019)	Anti-semitism or anti-homosexual speech (Leets 2002)

### Definition and operationalization of extremist discourse

Until now we have presented a distinction between the concepts of radicalization and extremism, choosing the latter as a key concept to justify the aims of this article. Also, extremism has been compared to other concepts that tend to appear in related studies. As has been stated, this term can have different meanings depending on the approach considered by the author, and this is why its relevant to establish a clear definition to depart from. In this review, our definition of extremism will be “an ideological movement, contrary to the democratic and ethical values of a society, that uses different methods, including violence (physical or verbal) to achieve its objectives”.

Following this definition, a second step would be to clarify what this article refers to as *extremist discourse*. While it could be conceived as “the use of language held by people when expressing their extremist views”, several authors have highlighted several features that characterizes an extremist narrative from a regular discourse. These features, derived from different works (Ashour 2010; Bennett Furlow and Goodall 2011; Fortuna and Nunes 2018; Sakki and Pettersson 2016; Torregrosa et al. 2020), can be summarized as follows:

- Types of extremist narrative: there are several ways by which extremist narratives justify their vision and objectives. Ashour (Ashour 2010) divided these narratives into five categories: political, historical, socio-psychological, instrumental and theological/moral:
  - Political: the discourse includes references to grievances from one or more groups towards other groups.
  - Historical: legitimization of the political grievance narratives through the use of historical examples and similes.
  - Socio-psychological: glorification of acts against the system, either violent or not.
  - Instrumental: justification of the violence and “self-defense” as a way towards reaching objectives.
  - Theological/moral: legitimization of actions or reactions against political grievance or social oppression through religion, morality and/or ethics.
- Linguistic style: the narrative styles or topics mentioned previously build upon a specific vocabulary and style that help extremists structure their discourse. Several articles have unveiled differences on the linguistic style from radical and extremist texts compared to a regular sample of texts (Cohen et al. 2014). For example, the higher use of first and third person plural pronouns, a more negative tone or the use of more words related to negative topics are common to these texts (Torregrosa et al. 2020).
- Use of discursive resources such as hate speech, otherness or war narrative: extremist texts tend to use discursive resources to convey their actions and ideas towards others. Some of these techniques have been studied in depth, such as hate speech (Fortuna and Nunes 2018), otherness (Sakki and Pettersson 2016) or the use of war terminology to create “enemies” and to communicate a “call to action” to others (Bennett Furlow and Goodall 2011).

In this vein, both the definition and operationalization of extremist discourse have been stated. This type of discourse is characterized by the use of specific narratives, an aggressive and polarized linguistic style and several techniques oriented to justify a feeling of superiority or inferiority towards another group. Considering this, NLP techniques can be exploited to analyze texts and detect and describe useful insights in order to determine when a user is holding this type of discourse. The next sections of this review will elaborate on how the community has used NLP to analyze extremist discourse on Social Media, and the outcomes reported in the reviewed studies.

## Methodology

This section describes the process carried out to conduct the survey of the articles that apply NLP to extremism research. This process was conducted through a systematic approach, retrieving all the articles from four scientific databases: Scopus, ScienceDirect, IEEE Xplore and Web of Science.

The process to conduct the screening process and the review of the articles followed the next steps:

1. Search in the databases. Concerning the thesaurus used for the search, it was decided to use both the *extremism* and *radicalization* terms in the search queries. The reason for this decision was that, as stated before, it is quite common that authors use these concepts as synonyms (Bötticher 2017; Schmid 2013). Second, while the thesaurus “Natural Language Processing” was included, we also decided to extend the search with different subtopics, such as “Sentiment analysis”, “Topic detection” and “Semantic analysis”. Eventually, and due to the recent contributions from the field of deep learning to natural language processing (Young et al. 2018), the subtopic “Deep learning” was also added to the search. Therefore, the thesaurus finally included in the searching process is: *(“Natural Language Processing” OR “Sentiment Analysis” OR “Topic Detection” OR “Semantic Analysis” OR “Deep Learning”) AND (“Extremism” OR “Radicalization”)* No bounded time span was selected when conducting the review, so that the articles returned from the search can be published in any year. The extraction was conducted in January 2021, resulting in 729 documents from the different databases. Table 2 shows the distribution of articles found per queried database. After deleting duplicates and filtering out non-scientific articles (e.g. indexes), 675 articles remained in the literature corpus.
2. First screening: title, abstract and methodology. After the search process, an extensive screening of the articles was conducted, which consisted of checking the title, the abstract and the methodology to find out whether the retained articles met the inclusion criteria of the review. This criteria can be summarized as:
  - The documents must empirically apply NLP to extremism description or classification.
  - The analysis conducted on the documents must be quantitative.
  - The documents must clearly state the NLP techniques they use to conduct the analysis.
  - The documents must present a clear methodology, including all the scores and the process they followed to conduct the analysis.
  - The article must be written in English.
  After this general screening process, 70 documents were finally held for further review.
3. Second screening: article’s content. A second more exhaustive review over these articles was performed, carefully reading the content of each document and excluding those that were confirmed not to accomplishing the criteria presented above. After the second screening process, 6 additional articles were discarded. The remaining ones (a total of 64) were finally included for the review.
4. Analysis of the selected articles and input extraction: the final step of the review process was the analysis and systematic comparison of the outcomes obtained from the different articles regarding extremism and NLP. Next sections condense and summarize the information obtained from this process.

**Table 2:** Articles extracted from the different databases that apply NLP to extremism research

Data source	No. articles
ScienceDirect	95
Scopus	573
Web of Science	41
IEEE Xplore	20
Total	729

## General descriptive analysis of the articles

This section presents a general descriptive analysis of the articles finally included on the review. Firstly, a general introduction is presented where the publishing years and the types of extremism detected are reviewed. Then, to identify the most relevant topics related to NLP that deal with the selected articles, a textual analysis has been performed using their indexing keywords. This description will be also used to structure the following sections of the paper, as it shows a general picture about the main topics addressed by the reviewed contributions.

**Fig. 3:**
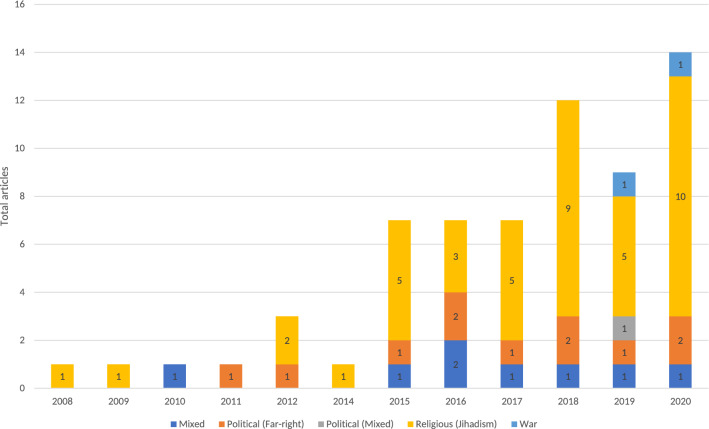
Type of extremism addressed by the articles included in the survey

Analyzing the timeline of the reviewed publications and the type of extremism under analysis, it can be observed that the interest in applying NLP to study extremism has been increasing sharply during recent years. This is shown in Fig. 3, which in turn supports the rationale and ideas given in the introduction of this review: most articles were published during or after 2015, which overlaps the time lapse when ISIS was more active.

Besides, as stated in Fig. 3, the most frequently addressed type of extremism in the reviewed articles is jihadi extremism, with a significant gap to the rest of types. In general terms, there are 5 types of extremism approached in the literature: religious (all of them concerning jihadism), political (far-right) political mixed (concerning far right/far left), war (concerning conflicts in different countries, such as Afghanistan), and mixed (studying both religious and political extremism). Since 2015, the number of works that use NLP to identify extremism have substantially increased. In this last period, while jihadi extremism has attracted more interest, political extremism remains relatively steady. Therefore, it can be concluded that the two predominant types of extremism have been the religious and political ones.

**Fig. 4:**
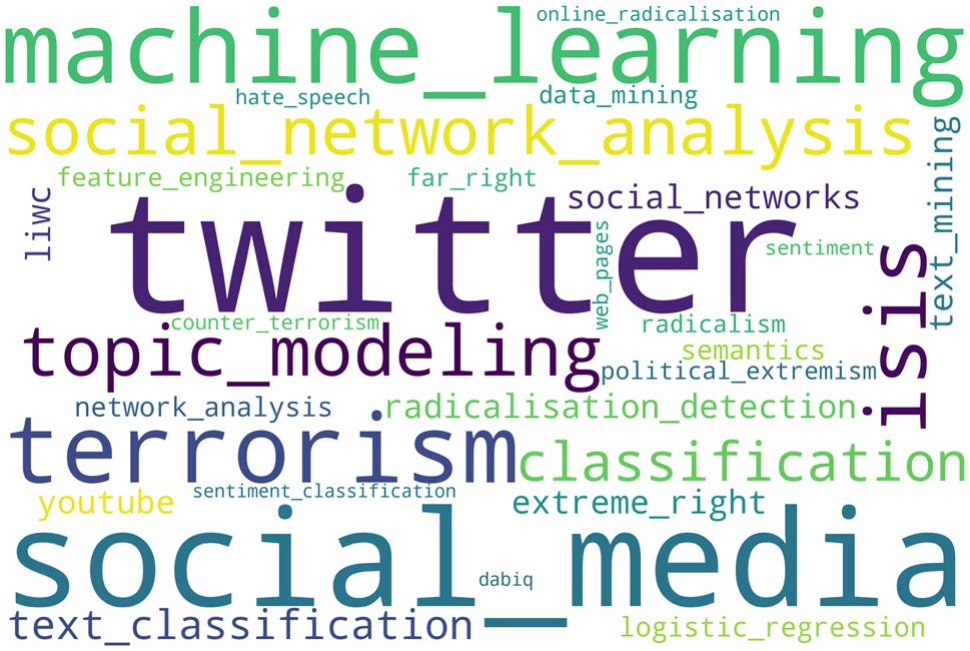
Word cloud of keywords extracted from the analyzed articles

We proceed with our preliminary analysis to determine the more common topics associated with the thesaurus used in the search for the articles. To this end, a textual analysis of the keywords related to the reviewed articles has been performed. For this purpose, Fig. 4 depicts a word cloud with the top 30 of the most frequently used keywords by the articles (keywords used as thesaurus were excluded from the count). As can be seen, keywords can be grouped under 4 similar directions:

1. The different NLP approaches in use (e.g. topic modeling, sentiment classification or semantics).
2. The source of the analyzed data (e.g. Twitter, social media, YouTube, web pages, or Dabiq, a jihadi magazine), as well as specific tools that can be used (e.g. Linguistic Inquiry Word Count).
3. Different key terms related to extremism (e.g. terrorism, ISIS, far-right, extreme right, hate speech, online radicalization, or radicalism).
4. The applied methodology, including classification techniques (ML, classification, logistic regression or feature engineering).

It is relevant to note at this point that, while not the objective of this survey, social network analysis (SNA) appeared as one of the most used keywords (both social network analysis and related variants, such as Social Networks and Network Analysis) (Bello-Orgaz et al. 2016; Camacho et al. 2020), which emerges as one of the concurrent approaches when conducting NLP analysis.

## NLP techniques for extremism research

The main objective of NLP techniques is to transform free text into structured data by capturing its lexical, syntactic and semantic information to acquire or infer new knowledge. Considering this, the NLP process can be divided into two main phases:

1. Text preprocessing: simplifying and preparing the text for its further analysis.
2. Feature generation: transforming the text into a structured data representation suitable to be used by the different computational methods of analysis.

Text preprocessing techniques are quite acknowledged in NLP, and therefore their review is out of the scope of this paper. Any researcher interested in these techniques can further read in Indurkhya and Damerau (2010) and Aggarwal (2018).

After preprocessing the textual data, different text mining techniques are used to transform tokens into structured data by capturing its lexical, syntactic and semantic information. These structured data can be eventually used as input for the different algorithms to acquire or infer new knowledge.

**Table 3:** Summary of NLP techniques for feature generation used in the reviewed literature

Approach	NLP technique	Percentage use	Articles
Lexical or vectorial	N-grams	28.12%	de Pablo et al. (2020); Rehman et al. (2021); Sharif et al. (2019); Kinney et al. (2018); Masood (2021); Kim et al. (2017); Hartung et al. (2017); Saif et al. (2017); Ben-David and Fernández (2016); Prentice et al. (2012); Rekik et al. (2019, 2020); Fernandez et al. (2018); Sharif et al. (2020); Abd-Elaal et al. (2020); Kursuncu et al. (2019); Nouh et al. (2019); Hall et al. (2020)
	Dictionaries	37.5%	Scrivens et al. (2020); Alizadeh et al. (2019); Devyatkin et al. (2017); Mirani and Sasi (2016); Saif et al. (2016); Bisgin et al. (2019); Rowe and Saif (2016); Scanlon and Gerber (2015); Gomes et al. (2017); Johnston and Weiss (2017); Johnston and Marku (2020); Ottoni et al. (2018); Hall et al. (2020); Saif et al. (2017); Abdelzaher (2019); Dillon et al. (2020); Klein and Muis (2019); Owoeye and Weir (2018); Rekik et al. (2019, 2020); Torregrosa et al. (2020); Wei and Singh (2018); Fernandez et al. (2018); Smith et al. (2020)
	TF	50%	Abdelzaher (2019); Agarwal and Sureka (2015); Ben-David and Fernández (2016); Bisgin et al. (2019); Chen (2008); de Pablo et al. (2020); Dillon et al. (2020); Figea et al. (2016); Hartung et al. (2017); Kinney et al. (2018); Klein and Muis (2019); Macnair and Frank (2018); Owoeye and Weir (2018, 2019); Rekik et al. (2019, 2020); Rowe and Saif (2016); Scanlon and Gerber (2015); Scrivens et al. (2015, 2020); Torregrosa et al. (2020); Wei et al. (2016); Wei and Singh (2018); Alizadeh et al. (2019); Fernandez et al. (2018); Smith et al. (2020); Bermingham et al. (2009); Araque and Iglesias (2020); Kursuncu et al. (2019); Prentice et al. (2012); Alghamdi and Selamat (2012); Devyatkin et al. (2017); Stankov et al. (2010)
	TF-IDF	23.43%	Alghamdi and Selamat (2012); Ahmad et al. (2019); Heidarysafa et al. (2020); Mariconti et al. (2019); O’Callaghan et al. (2012); Rehman et al. (2021); Sabbah and Selamat (2015); Sharif et al. (2019, 2020); Yang et al. (2011); Zahra et al. (2018); Abd-Elaal et al. (2020); Kim et al. (2017); Masood (2021); Nouh et al. (2019)
	Dichotomous appearance	1.56%	Wadhwa and Bhatia (2015)
	Log-likelihood	3.12%	Stankov et al. (2010); Prentice et al. (2012)
Neural language models	Word2Vec	9.37%	Abd-Elaal et al. (2020); Araque and Iglesias (2020); Johnston and Marku (2020); Kim et al. (2017); Kursuncu et al. (2019); Masood (2021); Nouh et al. (2019); Ottoni et al. (2018)
	FastText	4.68%	Ahmad et al. (2019); Araque and Iglesias (2020); Devyatkin et al. (2017)
	GloVe	3.12%	Araque and Iglesias (2020); Gomes et al. (2017)
Sintantic and semantic	Part-of-speech	25%	Devyatkin et al. (2017); Owoeye and Weir (2018); Mariconti et al. (2019); Masood (2021); Wignell et al. (2018); Macnair and Frank (2018); Figea et al. (2016); Skillicorn (2015); Scrivens and Frank (2016); Scrivens et al. (2018); Weir et al. (2016); de Pablo et al. (2020); Owoeye and Weir (2019); Scrivens et al. (2015); Sikos et al. (2014); Yang et al. (2011)
	NER	7.81%	Bisgin et al. (2019); Saif et al. (2017, 2016); Fernandez and Alani (2018); Hartung et al. (2017)
	LSF	4.68%	Kim et al. (2017); Masood (2021); Hartung et al. (2017)
	Parse trees	1.56%	Sikos et al. (2014)
	LDA	15.62%	Bisgin et al. (2019); Scanlon and Gerber (2015); Ottoni et al. (2018); Hall et al. (2020); Saif et al. (2017); Kursuncu et al. (2019); Heidarysafa et al. (2020); Alizadeh et al. (2019); Kinney et al. (2018); Kim et al. (2017)
	NMF	4.68%	Heidarysafa et al. (2020); O’Callaghan et al. (2015, 2012)
	Sentiment scoring	37.49%	Wignell et al. (2018); Chen (2008); Saif et al. (2017); Hartung et al. (2017); Masood (2021); Heidarysafa et al. (2020); Hall et al. (2020); Owoeye and Weir (2018); Macnair and Frank (2018); Figea et al. (2016); Scrivens and Frank (2016); Scrivens et al. (2018); Weir et al. (2016); Owoeye and Weir (2019); Scrivens et al. (2015); Mirani and Sasi (2016); Rowe and Saif (2016); Dillon et al. (2020); Torregrosa et al. (2020); Araque and Iglesias (2020); Scrivens et al. (2020); Wei et al. (2016); Bermingham et al. (2009); Ahmad et al. (2019)
	Semantic tagging	12.50%	Wignell et al. (2018); Saif et al. (2017, 2016); Fernandez and Alani (2018); Ottoni et al. (2018); Devyatkin et al. (2017); Abdelzaher (2019); Prentice et al. (2012)
	Word/sentence length	7.81%	Stankov et al. (2010); Yang et al. (2011); Sikos et al. (2014); Weir et al. (2016); Scrivens et al. (2018)
	Use of emoticons	3.12%	Agarwal and Sureka (2015); Wei et al. (2016)
	Use of punctuation	3.12%	Sikos et al. (2014); Yang et al. (2011)

Table 3 presents all the techniques mentioned on the review, together with the articles included on the review that have been applied them as part of their methodological approach. These techniques can be grouped into three different categories according to the type of captured linguistic information, which are explained below in detail in the following subsections. A first descriptive analysis of the techniques is conducted for each of these subsections. Afterwards, a comparative analysis of these techniques is carried out within the area of extremism research, stressing on the advantages and disadvantages of each technique within this specific domain.

### Lexical or vectorial based features

The tokens extracted from the preprocessing phase have to be transformed into more complex data structures representing a final textual features to be further processed. For this purpose, different techniques of text representation modeling can be applied. Vector space models (VSM) (Turney and Pantel 2010) is one of the most widely text representation used in classical NLP approaches. The idea of the VSM is to represent each text or document as a set of points in a space (a vector in a vector space) based on the token extracted. After the tokenization process, the first step to generate this type of representation consists of defining the weighting technique to compute the tokens (terms) appearance’s frequency in a text. The articles reviewed mention several different techniques to generate this vector representation:

- N-grams: tokens of size 1 are obtained from preprocess the free texts, which means that represents only one word. However, sentences generally contain compound terms (such as living room or coffee machine) formed by several words with a single meaning. The use of grouping multiple tokens together to represent that inherent meaning can be very beneficial for subsequent NLP tasks. This is indeed what n-grams models enable (Sidorov et al. 2012). A uni-gram is any single element of the text, whereas a bi-gram or a tri-gram is composed by two or three elements, respectively, which appear sequentially on the text. Skip-gram is a special version of n-gram: it works in the same way, but considering tokens that are not necessarily juxtaposed in the text. Therefore, an analysis based on n-grams considers *n* elements as a single token. One of the main advantages of this approach is that high “*n*” sizes provide contextual information for words (Fortuna and Nunes 2018). Table 4 summarizes which type of n-grams are in use in the reviewed articles, where unigrams are not shown since, as mentioned above, they would be 1-sized tokens that are already elicited by preprocessing techniques.
- Dictionaries: they are pre-established lists of lexicons (words or sentences) used for filtering or grouping the preprocessed tokens. Therefore, any term found inside the lexicon is considered as a final token to generate the final text representation. Dictionaries can also group the frequency of terms as a whole token, thus calculating the frequency of occurrence of a dictionary itself. The main advantage of the dictionaries is that they capture concepts defined by different terms. By contrast, they are also very vulnerable to words that are not previously included in the lexicon and to the continuous change of language.
- Term frequency (TF): is the more basic weighting technique in NLP, and consists of the raw sum of the occurrence of each token found in the text. It can be represented as *tf(t, d)*, wherein *t* denotes the number of times a token appears in document *d*.
- Term frequency-inverse document frequency (TF-IDF): it is an evolution of the aforementioned TF. While the TF just sums the frequency of occurrence of a token in a text, TF-IDF also divides it by the frequency of occurrence of a word in the whole corpus. When a word is more frequent in a text than in the set of texts, it means that this word is relevant for the text, and therefore it is given a higher score. It is useful for discriminating between relevant words and words with no relevant meaning, such as stop-words (Fortuna and Nunes 2018).
- Dichotomous appearance: it represents the presence or absence of a token. Therefore, it is computed as 0 if the term does not appear, and 1 if the term appears.
- Log-likelihood (Dunning 1993): it is used to compute the significance of the co-occurrence of two variables (for example, two tokens or a token with the group used for classification). Therefore, this technique does not account for the frequency of a single token, but for the frequency of two conditions appearing together, which may include one or two tokens.

**Table 4:** Type of n-gram model used in the reviewed articles

N-gram type	Percentage use	Articles using it
Bi-gram	15.62%	de Pablo et al. (2020); Rehman et al. (2021); Sharif et al. (2019); Kinney et al. (2018); Masood (2021); Kim et al. (2017); Hartung et al. (2017); Saif et al. (2017); Ben-David and Fernández (2016); Prentice et al. (2012)
Bi-gram + Tri-gram	6.25%	Rekik et al. (2019, 2020); Fernandez et al. (2018); Sharif et al. (2020)
Bi-gram + Tri-gram + Skip-gram	4.68%	Abd-Elaal et al. (2020); Kursuncu et al. (2019); Nouh et al. (2019)
Tri-gram + Tetra-gram + Penta-gram	1.56%	Hall et al. (2020)

Focusing on Table 3, the first point to be highlighted is the high use of n-grams and dictionary techniques, exceeding 25% in both cases. This is due to the fact that, from the text preprocessing phase, tokens of size 1 are obtained representing the text. In many cases, before applying more complex techniques that transform such tokens into complex data structures, it is beneficial to apply basic NLP techniques. These techniques allow grouping or filtering the tokens by aggregating them at a first level of lexical information.

The major advantage provided by the n-grams approach is that it is independent from the text. This means that all the text can be vectorized using these techniques, no matter if they appear on a lexicon or not. This is specially useful when applying NLP to extremism research, as texts usually combine terms in different languages. However, this versatility also poses a handicap: the vectorized terms may have no relevant meaning for the researcher, and therefore extra work must be conducted in those cases to identify which terms are relevant.

On the other hand, the use of dictionaries is helpful to detect and classify tokens into meaningful psycho-linguistic categories (Fernandez et al. 2018; Figea et al. 2016). This is a great advantage in the field of extremism research, taking into consideration the psychological background that motivates extremist behavior. In fact, one of the main dictionary based tool, Linguistic Inquiry Word Count, or LIWC (Pennebaker et al. 2001), was forged with the aim of conducting psychological research from texts. It has been frequently applied to extract psychological insights and extremist slang from extremist texts (Torregrosa et al. 2020). However, dictionaries require a previous effort from the researchers to prepare the lexicons or to adapt them to other languages (Sikos et al. 2014). This last point is specially relevant in the case of jihadi extremism, as texts usually combine Islamic terminology (written in Arabic) with different languages (Sikos et al. 2014).

Continuing with the analysis of the vectorial space models applied in the reviewed articles, TF and TF-IDF are the most used techniques. As stated previously, TF-IDF is an evolution from TF, using IDF to eliminate common terms from the text, leaving behind the less used terms, which can be relevant to discriminate textual patterns (in this case, extremist content). Taking into consideration that several articles from the review conduct filtering preprocessing techniques to eliminate irrelevant terms (such as stop-words), there is not a huge difference among them concerning the extremism research field. The main advantage of these techniques is their simplicity and broad use, which make them the most commonly applied techniques. Unfortunately, they have a great disadvantage: they do not provide semantic information about the terms.

Dichotomous appearance was only used in one article. While it presents a clear advantage (it is straightforward to implement), it has one main disadvantage: as stated in the previous section, some terms are used with different semantic meanings in regular and extremist texts (Fernandez and Alani 2018; Gomes et al. 2017; Saif et al. 2016; Wei and Singh 2018). Analyzing only the occurrence of a term can be poorly informative for the model. Finally, log-likelihood can be used for analysing association among terms, which allows providing more contextual information. However, it is still a very scarcely utilized technique within the extremist field of study. A brief summary of the advantages and disadvantages of all these techniques appears in Table 5.

**Table 5:** Comparison of vector space model based techniques to generate features in the reviewed articles

Technique	Advantages	Disadvantages
N-grams	Able to keep semantic information	Captures basic semantic information
	High versatility, due to its independence from the text (useful for multi-language texts)	The tokens detected may not have interest for the researcher
Dictionaries	Useful to conduct psycho-linguistic meaningful analysis	Low versatility (vulnerable to changes on the language and word structure)
	Useful to detect and classify specific slang and terminology	Highly dependent on the lexicons included
TF/TF-IDF	Simple and widely used	Not capture semantic context information
		TF needs a previous stop-words filtering
Dichotomous appearance	The simplest technique	Does not capture semantic context information
Log likelihood	Captures information of association among terms	Few applied in the area information

### Neural language models (word embedding)

Techniques based on neural models include a set of methods that transform tokens obtained from the preprocessing phase into meaningful vectors through the use of neural networks, allowing to capture the relationship among them (Levy and Goldberg 2014) and, therefore, information about words semantically related. In recent years, the application of these models in the field of extremism research have gained an increasing relevance, as they are useful for retaining information about the semantic meaning of the terms. This is precisely the advantage of this type of models to extract textual features compared with the classics models seen in the previous section. This aspect is specially relevant when applied to classification tasks and the use of *deep learning* to identify extremist content (Johnston and Marku 2020; Johnston and Weiss 2017). The most common neural models identified throughout the reviewed literature are:

- Word2Vec: it allows predicting words depending on the context, maintaining the semantic meaning of the sentence. To this end, the model creates a vector related to each word through the use of a single-layer neural network, which can be interpreted as a space embedding. Words that are more likely to appear together in the text will be mapped closer in that space, therefore sharing semantic context (Mikolov et al. 2013). Among the different versions of this technique, the continuous Bag-of-Word model and the Skip-Gram model are arguably the most commonly used ones (Goldberg and Levy 2014; Rong 2014).
- FastText: developed by Facebook (Bojanowski et al. 2017), it operates similarly to Word2Vec skip-gram, but overcoming two limitations of this model: it incorporates subwords in the embedding process, thereby permitting to include words that were not contained in the original lexicon (Schmitt et al. 2018).
- GloVe: developed at the Stanford University (Pennington et al. 2014), it relies on the use of a word co-occurrence matrix, to which factorization techniques are applied towards extracting the vectors associated to each word. While Word2Vec reportedly has a better performance than this technique, Glove has the advantage of having more available trained models to work with (Mikolov et al. 2017).

After analysing the usage of these approaches in the reviewed literature on extremism, four different purposes of word embedding methods can be discriminated:

1. To conduct bias analysis (how pejorative terms are related to some entities and not to others) (Ottoni et al. 2018).
2. To check how two texts use similar tokens but with different meanings (Gomes et al. 2017; Kursuncu et al. 2019).
3. To create new lexicons based on an already checked text (Araque and Iglesias 2020; Nouh et al. 2019).
4. To overcome language limitations on extremist detection (Johnston and Weiss 2017).

Regarding the frequency of use of these techniques in the field of extremism, Table 3 reveals that the most used technique (Word2Vec) does not reach 10%, a value much lower than most of the classical techniques based on vector space models. This is due to the fact that this type of approach is becoming of great importance just in the last few years, and it is at the current time when its application to the field of extremism research is growing in momentum.

Only one article reported a comparison among FastText, Word2Vec and GloVe within an extremism classification task. FastText performed slightly better than the other two. However, Word2Vec and its variations, such as doc2vec (Lau and Baldwin 2016) or graph2vec (Narayanan et al. 2017), still outstand as the most resorted pre-trained word embeddings. Table 6 summarizes the comparison among these techniques in the context of extremism research. A brief summary of the advantages and disadvantages of all these techniques is also given in the same table.

**Table 6:** Comparison of neural techniques to generate features used in the reviewed articles

Technique	Advantages	Disadvantages
Word2Vec	Allows predicting words depending on the context	Does not recognize words not included in the trained lexicon (problematic in multilingual approaches)
FastText	Allows incorporating words not contained on trained lexicon	Few applied in the area
GloVe	High amount of trained models to work with	Scarcely applied in the area

### Syntactic and semantic features

Some NLP techniques rely on the analysis of data according to a particular context for generating features representing the text (Krippendorff 2018). The type of contextual information depends on the NLP technique under consideration, but common approaches include sentiment analysis, topic detection or semantic analysis, among others. Techniques of this type used by the reviewed articles include:

- Part-of-speech (POS): it allows tagging every word with its grammatical category (e.g. nouns, verbs or adjectives) depending on the structure of the text where it is found (Cutting et al. 1992).
- Lexical syntactic feature-based (LSF): it allows capturing the dependence inside a sentence or a text between two terms (Benito Sánchez 2019). These two terms are later compared to determine the context and the direction of the expression.
- Named entity recognition (NER): it deals with the identification of entities (e.g. names, organizations or locations) in the text, tagging them as relevant subjects (Ritter et al. 2011).
- Parse trees (PT): it constructs a representation of how the concepts can be used recursively in a sentence. Parse trees include all the tokens and their relationships, along with a set of rules that allows substituting the token while maintaining the syntactic rules.
- Latent Dirichlet allocation (LDA): it is one of the most popular NLP techniques for topic detection. It extracts topics from a corpus of text based on word probabilities: for each latent topic, it extracts the probability distribution of a combination of words, which helps identify the main topics. (Jelodar et al. 2019).
- Non-negative matrix factorization (NMF): it is a topic modeling technique which relies on the use of linear algebra algorithms in a TF-IDF document matrix to define topics (Chen et al. 2019).
- Sentiment scoring (SS): it provides a score for every text unit (e.g. sentence or text) based on its latent emotional valence, with the aim of understanding the authors opinion or emotional state about something (Liu 2020). This score can be computed as dimensional (through a single scoring about the valence) or categorical (specifying which emotions are expressed in the text). Table 7 summarizes how both approaches are distributed among the reviewed articles.
- Semantic tagging (ST): it implies the process of automatically extracting concepts, entities or topics from the tokens in a text, which can be realized by assorted algorithmic means (e.g. Jovanovic et al. 2014).
- Word/sentence length: it analyzes the length of the words (based on characters) and/or the sentences (based on words) (Stankov et al. 2010; Yang et al. 2011; Sikos et al. 2014; Weir et al. 2016; Scrivens et al. 2018).
- Use of emoticons: emoticons are graphical figures to express emotions or behaviors on the text, using a combination of characters (Agarwal and Sureka 2015; Wei et al. 2016).
- Use of punctuation: this approach involves the analysis of the use of punctuation signs as part of the syntactic distribution of the sentence (Sikos et al. 2014; Yang et al. 2011).

**Table 7:** Type of sentiment analysis approaches using in the reviewed articles on extremism

Sentiment analysis approach	Percentage use	Articles using it
Sentiment scoring (dimensional)	32.81%	Wignell et al. (2018); Owoeye and Weir (2018); Scrivens et al. (2015); Hall et al. (2020); Chen (2008); Macnair and Frank (2018); Figea et al. (2016); Scrivens and Frank (2016); Scrivens et al. (2018); Weir et al. (2016); Owoeye and Weir (2019); Mirani and Sasi (2016); Rowe and Saif (2016); Dillon et al. (2020); Torregrosa et al. (2020); Scrivens et al. (2020); Wei et al. (2016); Bermingham et al. (2009); Masood (2021); Saif et al. (2017); Ahmad et al. (2019)
Emotion scoring (categorical)	9.37%	Wignell et al. (2018); Chen (2008); Heidarysafa et al. (2020); Araque and Iglesias (2020); Hartung et al. (2017); Ahmad et al. (2019)

These types of techniques go a step further into text representation, taking advantage of the tokens to conduct a more complex analysis. This is specially useful in the extremism research field, where simple term analysis or frequency can be misleading in the interpretation of outcomes, due to the disparity of semantic meaning behind the same term used by extremist and non-extremist groups (Fernandez and Alani 2018).

The first four aforementioned techniques (namely, POS, NER, LSF and PT), are used to analyze, tag and extract information about the syntactical structure underlying tokens. While POS tags each word with its syntactic function inside a sentence, NER is used to identify the nouns and entities present on the text. Then this information is used to determine which nouns from the text are actual people, organizations or locations (Hartung et al. 2017; Saif et al. 2017, 2016; Fernandez and Alani 2018; Bisgin et al. 2019), among others. In particular, according to the articles reviewed in our survey, NER evidenced that using a combination of noun semantic categories was statistically more accurate to determine if a text included extremist content than using token analysis, sentiment or topic features (Saif et al. 2017, 2016). Analyzing the frequency of application shown in Table 3, among these 4 techniques, the most commonly used in the field of extremism is POS (25%), being the rest of techniques used less frequently.

On the other hand, LSF and PT regard the syntax and the dependencies among tokens. In this case, LSF analyzes the relationship between two syntactically dependent tokens (Kim et al. 2017; Masood 2021), while parse trees build representations of several tokens and use their syntactic structure to find tokens combined in the same way (Sikos et al. 2014). LSF was compared to vectorial space models as classification feature, but it did not perform any better than the latter (Hartung et al. 2017).

In what refers to topic extraction, LDA and NMF have been the techniques of choice in many reviewed articles. LDA has the advantage of hinging on a statistical base and to be commonly used in the NLP literature (Heidarysafa et al. 2020). However, as stated in Alizadeh et al. (2019), it performs poorly with short texts (e.g. tweets). Taking into account that most of the articles reviewed use Twitter to extract their extremist datasets, this is an important disadvantage. NMF emerges as an alternative to LDA, presenting more readily interpretable results (O’Callaghan et al. 2012, 2015), and featuring a better performance over short texts (Chen et al. 2019). Notwithstanding these benefits, in the reviewed articles NMF is used much less frequently than LDA (see Table 3).

Adding a topic an “valence score” can help compose a representative idea about the author’s agreement with that topic (Bermingham et al. 2009; Scrivens et al. 2018). For example, two studies focused on Arabic regular population found out that Twitter users’ tone was more negative when ISIS committed a murder, won a battle or made a public call or movement (Mirani and Sasi 2016; Ceron et al. 2019). Sentiment scoring techniques are divided in two different approaches: a dimensional approach, based on a single score, and a categorical approach, based on the classification of tokens inside one or more emotions (such as anger, fear or happiness). A combination of both strategies can be found in some of the articles (Wignell et al. 2018; Figea et al. 2016). These techniques can be employed to measure the emotions expressed in the text, together with the opinion of the writer towards a specific token in the text (Bakshi et al. 2008). The main difference among them is their theoretical basis, but also the way they are applied: dimensional scoring usually involves selecting a token, around which the scoring process takes part. On the other hand, categorical scoring usually classifies tokens depending on the emotion they represent, and therefore are more focused on single tokens. In the case of extremism research, both approaches can be useful, as they can identify how extremist texts approach different topics (Wignell et al. 2018; Macnair and Frank 2018), the valence of their tones (Wei et al. 2016) or the connotations of the terms they use (Chen 2008). Finally, the concept of semantic tagging was used in the reviewed literature to tag tokens with semantic information regarding their context. This strategy, which is very similar to NER (sometimes using it), tags the tokens with entities, but also with concepts and categories (Wignell et al. 2018). Focusing on the use of this type of techniques in the reviewed articles, Table 3 shows that the sentiment analysis techniques are the most used within the techniques to extract syntactic and semantic features, exceeding 37% in the case of sentiment scoring.

The last three techniques elaborate on the analysis of the text formatting characteristics, to build other types of features that capture more information than that provided by the text itself. For example, the length and quantity of texts, sentences or words, the number of characters inside a word, the use of punctuation or emoticons. In all these cases, text characteristic features have been used as a complement to other text features, never as single features extracted from the free text. However, they have shown little impact when describing or predicting extremism in texts, and in general are applied in a marginal fraction of the reviewed works (as can be seen in the last 3 rows of Table 3).

Table 8 presents a summary of all the techniques used to generate syntactic and semantic features showing their advantages and disadvantages both in general application and in extremism literature.

**Table 8:** Comparison of syntactic and semantic based techniques to generate features for text representation

Technique	Advantages	Disadvantages
POS	Allows to detect the grammatical type of tokens	Regarding nouns, not as informative as NER
	Widely used in the area with different applications (term disambiguation or classification)	
NER	Detects entities, categorizing them. Useful to identify the main actors in an extremist discourse	Not as extended as POS, limited to nouns and to a trained lexicon
LSF	Provides a meaningful relationship among tokens.	Does not perform better in the applications within the area than more simple features
PT	Finds sentences with a grammatically similar structure	Does not inform about the tokens itself. Not commonly used on extremism literature
LDA	Widely used on extremism research	Performs poorly in short texts, such as tweets (very used to conduct extremism analysis)
	Performs closer to a human topic classifier than other techniques	Tends to over-generalize topics
NMF	Alternative for LDA showing a good performance over short texts.	Not commonly used by authors, who tend to use LDA
SS (Dim.)	Simple way of measuring a sentence emotional value	Does not provide elaborate information about emotions in the sentence
	Useful to detect opinions, specially useful when combined with the detection of entities in the radical discourse	
SC. (Cat.)	Provides information about emotions in the sentence, tagging tokens and sentences with emotional categories (Happiness, sadness, anger...)	Not so useful to detect opinions or tone towards a token
ST	As an evolution of NER, this approach “tags” nouns with their entity, concept and category	Useful to discriminate a word thanks to its context, very useful on extremism research
Text formatting	Captures more information than those provided by the text itself	Has to be used as a complement to other text features

## Applications of NLP in extremism research

The previous section has detailed all NLP techniques used in the reviewed works on extremism to process text and generate features as structured data. Depending on the objectives to be achieved in each of the reviewed works, one or several of these generated features are used to acquire new knowledge. In general two main purposes have been identified in the reviewed papers for which they are used:

1. As the input of classification models generated with ML algorithms to discriminate between extremist and non-extremist content.
2. To conduct a descriptive analysis characterizing the extremism: for example, to detect slang that is specific of extremism.

Based on these two main approaches, the next subsections present a descriptive and comparative analysis of the works that undertake each of these purposes, pausing and examining their main outcomes.

### Classification approaches

**Fig. 5:**
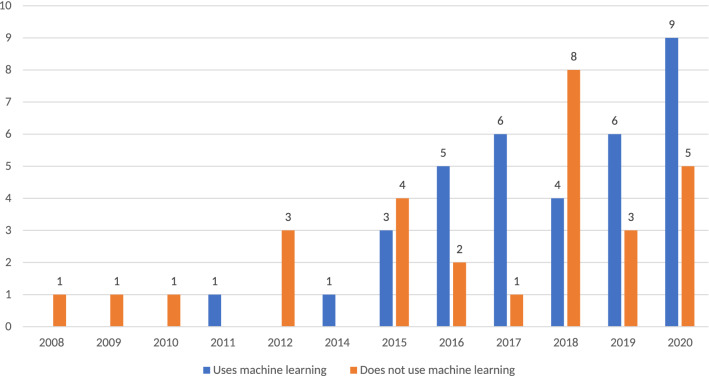
Frequency of articles using classification techniques versus those not using them

As can be derived from the general analysis of the reviewed articles presented in Sect. 4, classification is one of the main topics of interest regarding NLP applications on extremism. This is not surprising, as one of the key objectives of this research field is to help law enforcement agencies identify extremist content. More than half of the articles included in the review (54.68% of the articles) made use of one or more classification algorithms, specially during the first years of ISIS activity. As shown in Fig. 5, 2015 and 2018 were the only years after the beginning of ISIS activity in which there are more articles not resorting to classification techniques than articles using them. The common use of classification approaches shows that there was a higher interest in detecting extremism than in defining it.

**Fig. 6:**
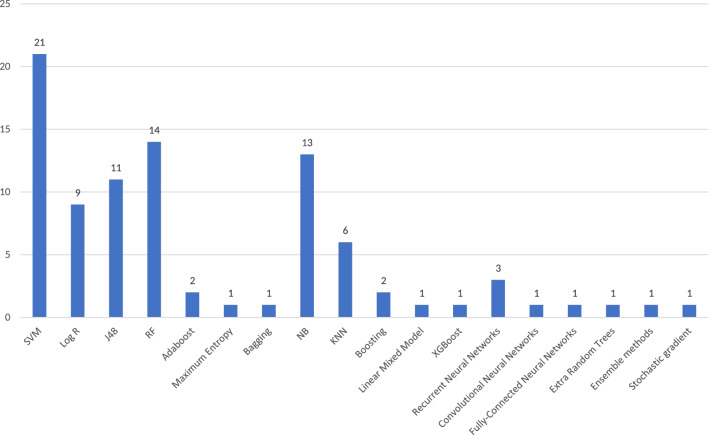
Type of ML model used in the literature related to extremism research

With the goal of training classification models based on NLP features to discriminate between extremist and non-extremist content, different ML algorithms have been applied in the reviewed works. These works uses ML models to address issues that goes from sentiment analysis (using a pre-labelled dataset) to proper user classification (extremist vs non-extremist). Figure 6 illustrates the frequency of application of every ML algorithm in the articles under review, where it can be noted that support vector machine (SVM) is the most widely considered model, followed by random forest, Naïve Bayes and decision tree (J48).

Regarding the model used by each article, Table 9 summarizes what kind of ML algorithms were used by all the articles including classification tasks. It also highlights the NLP features that are directly (or indirectly) involved in the generation of the classification models.

Apart from these classification tasks, five articles conducted other predictive learning tasks. These include the prediction of how the radicalization process takes place (Fernandez et al. 2018), how extremist behavioral changes occur among the members of a group (Smith et al. 2020), the daily level of online recruitment activities conducted by extremist groups (Scanlon and Gerber 2015), the risk of a video to be raided by extremist groups (Mariconti et al. 2019) or the risk of pro-ISIS terms as part of a person’s vocabulary (Rowe and Saif 2016).

We first place our attention on the use of basic features based on vectorial space models, such as n-grams and dictionaries (shown in Table 9). The first ones (n-grams) (Bisgin et al. 2019; Hartung et al. 2017; Kursuncu et al. 2019; Owoeye and Weir 2018; Rekik et al. 2019; Scanlon and Gerber 2015; Sharif et al. 2019; Zahra et al. 2018) have been used more than the second ones (dictionaries) (Ahmad et al. 2019; Araque and Iglesias 2020; Fernandez et al. 2018; Kursuncu et al. 2019).

Nevertheless, it is difficult to determine which of these two techniques performs best. In fact, the study of Figea et al. (2016) found out that there is no relevant difference between using dependent techniques (such as n-grams) or independent (such as LIWC) from the text when creating a classification model. A general limitation from both techniques is that similar terms can be used with different meanings in two texts, leading to confusions during the data interpretation process (Saif et al. 2016; Fernandez and Alani 2018; Wei and Singh 2018; Gomes et al. 2017. This is common in the context of religious radicalization, where religious terms can be used by regular religious texts, but also by extremists texts (Gomes et al. 2017). Although the use of n-grams is a possible way to overcome this limitation, they are a primitive option to keep semantic information (Hall et al. 2020; Sharif et al. 2019). Nonetheless, there are techniques that are more informative than these ones when conducting complex NLP analysis. For example, n-grams were reported to perform worse when identifying topics in radical texts than LDA or dictionaries (Hall et al. 2020).

**Table 9:** Type of features input to the ML models employed in the reviewed articles

ML method	Features												
	N-grams	Dic.	TF-IDF	TF	POS	NER	LSF	PT	SS	LDA	Emb.	ST	Others
SVM	Hartung et al. (2017); Masood (2021); Saif et al. (2017); Rehman et al. (2021); Sharif et al. (2019); Abd-Elaal et al. (2020)	Figea et al. (2016); Sikos et al. (2014); Yang et al. (2011); Agarwal and Sureka (2015); Rehman et al. (2021)	Yang et al. (2011); Rehman et al. (2021); Sharif et al. (2019); Masood (2021); Kim et al. (2017); Abd-Elaal et al. (2020)	Hartung et al. (2017); Masood (2021); Scanlon and Gerber (2015); Ahmad et al. (2019); Devyatkin et al. (2017); Kim et al. (2017); Mirani and Sasi (2016); Chen (2008); Figea et al. (2016); Wei et al. (2016); Agarwal and Sureka (2015); Araque and Iglesias (2020); Fernandez and Alani (2018)	Figea et al. (2016); Sikos et al. (2014); Yang et al. (2011); Devyatkin et al. (2017)	Hartung et al. (2017); Yang et al. (2011)	Hartung et al. (2017); Masood (2021); Kim et al. (2017)	Sikos et al. (2014)	Figea et al. (2016); Mirani and Sasi (2016); Wei et al. (2016); Masood (2021); Saif et al. (2017); Yang et al. (2011); Ahmad et al. (2019); Araque and Iglesias (2020); Hartung et al. (2017)	Saif et al. (2017); Scanlon and Gerber (2015); Kim et al. (2017)	Araque and Iglesias (2020); Masood (2021); Devyatkin et al. (2017); Kim et al. (2017); Abd-Elaal et al. (2020)	Saif et al. (2017); Fernandez and Alani (2018); Devyatkin et al. (2017)	Sikos et al. (2014); Yang et al. (2011)
KNN	Sharif et al. (2019); Abd-Elaal et al. (2020)	Agarwal and Sureka (2015)	Sharif et al. (2019, 2020); Abd-Elaal et al. (2020)	Ahmad et al. (2019); Wei et al. (2016); Agarwal and Sureka (2015)					Wei et al. (2016); Ahmad et al. (2019)				
NB	Masood (2021); Rehman et al. (2021); Sharif et al. (2019, 2020); Abd-Elaal et al. (2020)	Yang et al. (2011); Rehman et al. (2021); Fernandez et al. (2018)	Yang et al. (2011); Zahra et al. (2018); Rehman et al. (2021); Sharif et al. (2019); Masood (2021); Abd-Elaal et al. (2020)	Masood (2021); Scanlon and Gerber (2015); Ahmad et al. (2019); Saif et al. (2016); Devyatkin et al. (2017); Sharif et al. (2020); Wei et al. (2016); Fernandez et al. (2018); Kursuncu et al. (2019); Fernandez and Alani (2018)	Yang et al. (2011); Devyatkin et al. (2017)	Yang et al. (2011)	Masood (2021)		Wei et al. (2016); Masood (2021); Yang et al. (2011); Ahmad et al. (2019)	Scanlon and Gerber (2015)	Masood (2021); Devyatkin et al. (2017); Kursuncu et al. (2019); Abd-Elaal et al. (2020)	Saif et al. (2016); Fernandez and Alani (2018); Devyatkin et al. (2017)	Yang et al. (2011)
Boosting				Scanlon and Gerber (2015); Devyatkin et al. (2017, 2017)						Scanlon and Gerber (2015)	Devyatkin et al. (2017)	Devyatkin et al. (2017)	
J48	Sharif et al. (2019); Rekik et al. (2020); Sharif et al. (2020); Abd-Elaal et al. (2020)	Fernandez et al. (2018)	Sharif et al. (2019, 2020); Masood (2021); Abd-Elaal et al. (2020)	Sharif et al. (2020); Mirani and Sasi (2016); Owoeye and Weir (2019); Rekik et al. (2020); Owoeye and Weir (2018); Fernandez et al. (2018); Fernandez and Alani (2018)	Owoeye and Weir (2018)				Owoeye and Weir (2018); Scrivens and Frank (2016); Weir et al. (2016); Owoeye and Weir (2019); Mirani and Sasi (2016)		Abd-Elaal et al. (2020)	Fernandez and Alani (2018)	Weir et al. (2016)
RF	Masood (2021); de Pablo et al. (2020); Rehman et al. (2021); Sharif et al. (2019, 2020); Abd-Elaal et al. (2020); Nouh et al. (2019)	Figea et al. (2016); Rehman et al. (2021); Nouh et al. (2019)	Ahmad et al. (2019); Mariconti et al. (2019); Rehman et al. (2021); Sharif et al. (2019, 2020); Abd-Elaal et al. (2020); Nouh et al. (2019)	Masood (2021); Mariconti et al. (2019); Ahmad et al. (2019); Devyatkin et al. (2017); Sharif et al. (2020); Mirani and Sasi (2016); Figea et al. (2016); de Pablo et al. (2020); Kursuncu et al. (2019)	Figea et al. (2016); Devyatkin et al. (2017); de Pablo et al. (2020)		Masood (2021)		Figea et al. (2016); Weir et al. (2016); Mirani and Sasi (2016); Masood (2021); Ahmad et al. (2019); Nouh et al. (2019)		Masood (2021); Devyatkin et al. (2017); Kursuncu et al. (2019); Abd-Elaal et al. (2020); Nouh et al. (2019)	Devyatkin et al. (2017)	Weir et al. (2016); de Pablo et al. (2020)
Adaboost		Figea et al. (2016); Yang et al. (2011)	Yang et al. (2011)	Figea et al. (2016)	Figea et al. (2016); Yang et al. (2011)	Yang et al. (2011)			Figea et al. (2016); Yang et al. (2011)				Yang et al. (2011)
Log R	Masood 2021; Sharif et al. 2020; Abd-Elaal et al. 2020	Smith et al. (2020); Fernandez et al. (2018)	Sharif et al. (2020); Masood (2021); Abd-Elaal et al. (2020)	Masood (2021); Devyatkin et al. (2017); Sharif et al. (2020); Wei et al. (2016); Smith et al. (2020); Fernandez et al. (2018); Araque and Iglesias (2020)	Devyatkin et al. (2017)		Masood (2021)		Wei et al. (2016); Masood (2021); Araque and Iglesias (2020)		Araque and Iglesias (2020); Masood (2021); Johnston and Marku (2020); Devyatkin et al. (2017); Abd-Elaal et al. (2020)	Devyatkin et al. (2017)	
LMM		Smith et al. (2020)		Smith et al. (2020)									
XGBoost			Kim et al. (2017)				Kim et al. (2017)			Kim et al. (2017)	Kim et al. (2017)		
Maximum entropy				Mirani and Sasi (2016)					Mirani and Sasi (2016)				
Bagging				Mirani and Sasi (2016)					Mirani and Sasi (2016)				
RNN				Mariconti et al. (2019)					Ahmad et al. (2019)		Johnston and Marku (2020); Ahmad et al. (2019)		
CNN									Ahmad et al. (2019)		Ahmad et al. (2019)		
FCNN											Johnston and Weiss (2017)		
Extra random trees			Mariconti et al. (2019)	Mariconti et al. (2019)									
Ensemble methods	Sharif et al. (2019)		Sharif et al. (2019)										
SGD	Sharif et al. (2020)		Sharif et al. (2020)	Sharif et al. (2020)									

*SVM* support vector machine, *KNN* K-nearest neighbors, *NB* Naïve Bayes, *RF* random forest, *Log R* logistic regression, *LMM* linear mixed models, *RNN* recurrent neural networks, *CNN* convolutional neural networks, *FCNN* fully-connected neural networks, *SGD* stochastic gradient descent

Regarding sentiment features, they are not usually used as a single feature to detect extremist content, specially concerning political radicalization (Scrivens et al. 2015). While these features do not perform bad either and they, in fact, perform better than other less complex features (Ahmad et al. 2019), classification models trained with more features usually perform better than those who use only sentiment features (Weir et al. 2016; Hartung et al. 2017; Saif et al. 2017; Owoeye and Weir 2018, 2019; Araque and Iglesias 2020). In fact, those classifiers based on exclusively semantic features performed better than those based on strictly sentiment features (Saif et al. 2017; Araque and Iglesias 2020). For example, a study conducted by Weir et al. (2016) compared the usefulness of two classification tools, one based on sentiment features and the other using POS feature together with text formatting features such as number of sentences, average length or quantity of characters. The second showed a better performance, but it could be due to the high number of features used in it. Other three articles (Sikos et al. 2014; Yang et al. 2011; Stankov et al. 2010) also utilized text formatting features and other text features, as characteristics to describe and classify extremist content. None of them rendered a significant difference with respect to classifiers that only use features that extract information from the text itself. Contrarily, there are several works which claim that text formatting features (such as sentence length (Yang et al. 2011) or emoticons (Agarwal and Sureka 2015; Wei et al. 2016) constitute a good add-on for improving the accuracy of classification models.

Finally, the best classification outcomes are achieved by using features based on neural models (word embedding). The main purpose of most articles that use features based on Neural Language Models in classification tasks is the detection of extremist content. As other types of features, they are quite dependent on the type of ML algorithm at hand (Masood 2021; Kim et al. 2017; Johnston and Marku 2020; Devyatkin et al. 2017). Nevertheless, they perform specially well when combined with neural networks of different types (Ahmad et al. 2019; Johnston and Weiss 2017). Contributions embracing this type of textual representation as classification features concur in a similar conclusion: features based on neural models tend to outperform other classical features such as vectorial space models (Devyatkin et al. 2017; Kursuncu et al. 2019; Masood 2021) or syntactic and semantic features (Kim et al. 2017; Araque and Iglesias 2020). One article, however, pointed out that word embeddings perform poorly when compared to n-grams when dealing with short pieces of text (Abd-Elaal et al. 2020). As happened with other NLP features, combining word embedding based features with other types of features also gave rise to better classification outcomes than using them in isolation (Araque and Iglesias 2020; Nouh et al. 2019).

### Descriptive approaches

A second application of NLP techniques in extremism research stemming from our literature analysis is the characterization and study of the phenomenon of extremism from a descriptive point of view. Within these works, four different descriptive focus can be established:

- Terms: descriptive analysis on the terms commonly used by extremists. Stated differently, characterization of the type of extremist vocabulary.
- Topics: detection of the most common topics discussed by extremist texts.
- Sentiment: analysis of the sentiment and tone of an extremist discourse.
- Semantic: analysis of the contextual information around terms inside an extremist text.

Table 10 summarizes the type of descriptive analysis performed for each of the articles reviewed. The simplest descriptive approach focuses on the terms, whereas the inclusion of other approaches (topics, sentiment, semantic or punctuation) adds extra layers to the description of the discourse. This is why the terms approach is central in the literature related to extremism description. In addition, we notice that almost all the rest of descriptive analysis perform a prior term analysis, elucidating the complementary of all these approaches. Sentiment analysis is the only one that is occasionally performed in an independent fashion.

**Table 10:** Descriptive linguistic approach used by the reviewed articles

Descriptive linguistic approach	Percentage use	Articles using it
Terms	67.85%	Heidarysafa et al. (2020); Rekik et al. (2019); Kinney et al. (2018); Gomes et al. (2017); Torregrosa et al. (2020); Alizadeh et al. (2019); Bisgin et al. (2019); Hall et al. (2020); Stankov et al. (2010); Prentice et al. (2012); Ben-David and Fernández (2016); Alghamdi and Selamat (2012); Bermingham et al. (2009); Klein and Muis (2019); Abdelzaher (2019); Wei and Singh (2018); Wignell et al. (2018); Macnair and Frank (2018); Skillicorn (2015)
Topics	46,42%	Heidarysafa et al. (2020); Kinney et al. (2018); Alizadeh et al. (2019); Bisgin et al. (2019); Hall et al. (2020); Ben-David and Fernández (2016); Alghamdi and Selamat (2012); Bermingham et al. (2009); Klein and Muis (2019); O’Callaghan et al. (2012); Ottoni et al. (2018); O’Callaghan et al. (2015); Wadhwa and Bhatia (2015)
Sentiment	39.28%	Heidarysafa et al. (2020); Bermingham et al. (2009); Torregrosa et al. (2020); Wignell et al. (2018); Macnair and Frank (2018); Chen (2008); Scrivens et al. (2020); Dillon et al. (2020); Scrivens et al. (2018, 2015); Alizadeh et al. (2019)
Semantic	17.85%	Wignell et al. (2018); Ottoni et al. (2018); Gomes et al. (2017); Prentice et al. (2012); Abdelzaher (2019)
Punctuation	3.57%	Stankov et al. (2010)

Regarding the insights about extremism found in the reviewed works, Sects. 6.2.1 and 6.2.2 highlight the main observed patterns, classified by the two predominant types of extremism found in Sect. 4: religious (mostly focused on jihadism) and political (mostly focused on far-right movements).

#### Literature insights about religious extremism

The insights obtained from the comparison of the literature regarding religious extremism, focused on jihadism, can be divided in different sections:

- Terms: When centering the scope of the analysis on common terms used by religious extremism, the name “ISIS” was more mentioned by neutral users than by extremist users (Wignell et al. 2018; Gomes et al. 2017; Bisgin et al. 2019), who preferred the term “Islamic State” or “Caliphate”. The more frequent terms encountered in extremist texts analyzed in the articles were related to religious (e.g. Allah, Jihad or Islam) or geographical references (e.g. Syria, Raqqa, America or Iraq) (Wignell et al. 2018; Gomes et al. 2017; Wei and Singh 2018; Bisgin et al. 2019; Skillicorn 2015). The descriptive analysis of the text also unveiled the common use of specific slang terms, such as “Crusaders”, “Mujahideen” or “Abu” (Gomes et al. 2017; Wei and Singh 2018).
- Topics: Works carrying out a descriptive analysis based on the topics show that the most frequent topic related to Jihadi extremism was, unsurprisingly, religion (Scanlon and Gerber 2015; Bermingham et al. 2009; Kinney et al. 2018). The most easily identifiable topics in Jihadi magazines were war, geopolitics, religious speech, government and administration (Bisgin et al. 2019). Inspire (Al Qaeda’s magazine) was rather focused on conflict legitimisation and philosophy, while Dabiq and Rumiyah (ISIS magazine) were more focused on the geopolitical conflict (Kinney et al. 2018). Some of the topics, such as recruitment, are reportedly hidden among topics referring to religious and military aspects of the Syria conflict (Scanlon and Gerber 2015).
- Sentiment: Combining sentiment analysis and topic detection, jihadi women happen to be more extreme than men in their messages related to nearly every relevant topic (Bermingham et al. 2009). Concerning the magazines, most of their texts have a negative tone and recurrently embrace terms related to fear, except when they discuss about topics such as paradise or martyrdom (Wignell et al. 2018; Macnair and Frank 2018). Words such as Allah or Islamic State were also spotted to have negative connotations when analyzed through a sentiment analysis approach. Authors hypothesized that this might be due to their use as a justification of violent behaviors. A study concerning jihadi radical forums also uncovered that the most extremist texts scored more on negative dimensions, using violence and hate terms, than more moderate alternatives (Chen 2008). Finally, a study postulated that radical users that presented a good tone towards ISIS (on their tweets) showed in fact complicity with it (Wei et al. 2016).
- Semantic: While the descriptive term analysis provides a first insight, it shall be remembered that context can alter the meaning of a token (Wei and Singh 2018). From this perspective, articles focused on semantic discrimination allow checking how these keywords are used depending on the intention of the text. For example, Gomes et al. (2017) stated that the background of the terms “ISIS”, “Islamic” and “Syria” changes as per the origin of the text under analysis (neutral or extremist). A study delving into the divergences of the semantic meaning of words, conducted by Fernandez and Alani (2018), classified terms into different semantic groups (category, entity and type of entity). Similar words were found to be used differently by radical and non-radical users, including the name of radical groups. Entities were concluded to be a good way of discriminating the semantic meaning of a term. Finally, the study of Kursuncu et al. (2019) conducted a comparative analysis between extremist and non-extremist religious users. Their findings resolved that while both groups shared terminology when referring to the religious concept, the extremist group made use of much more terms related to radical Islamism and hate speech. This goes in line with the evidence that token analysis techniques combined with other strategies can be more informative than using them alone.

As can be supported by these insights, and taking into consideration the features of an extremist discourse presented in Sect. 2.3, Jihadi extremism possesses several of these features. Their use of specific slang and expressions, together with a negative tone, shows how they present a specific linguistic style. Also, they endow their discourses with a special emphasis on a theological and moral narrative, but also with the glorification of religious acts of violence against a common enemy (Western society and non-believers). It is difficult to determine how much of their use of war topics relates to a specific narrative or the geopolitical situation of the territories in which they operate. Nevertheless, it is fair to state that war (and its instrumentalization) is a key element in the construction of their narrative.

#### Literature insights about political extremism

Focusing now on the reviewed works that conduct a descriptive analysis of the terms most commonly used by far-right extremism, and following the same structure as with religious extremism, we find different insights:

- Terms: An article analyzing an Alt-right community (Torregrosa et al. 2020) reported that they used racist (BlackMagic, WhitesLivesMatter), anti-immmigration (BuildTheWall, IllegalAliens) supremacist (WhiteGenocide, WhitePeople, ChasingDownWhites) and anti-left (AntifaTerrorists) terms and hashtags in their tweets. This work also exposed the use of specific slang to refer to other racial minorities, such as “aliens” to refer to immigrants. Among a sample of videos massively attacked by far-right groups from 4chan,^1^ some of the most mentioned keywords were “black”, “police”, “white”, “shot”, “gun”, “world”, “war”, “American”, “government” or “law” (Mariconti et al. 2019). Other relevant keywords of far-right extremist groups include the mention of the numbers “14” (a reference to the “fourteen words”, one of the most popular white nationalist slogan, coined by David Lane, a member of the white supremacist terrorist group known as The Order: “We must secure the existence of our people and a future for white children” (Michael 2009) and “88” (meaning “Heil Hitler”, as the H is the 8th letter of the alphabet), but also to the genocide, nazism, anti-islamic and anti-jewish groups (O’Callaghan et al. 2012, 2015).
- Topics: the more common topics discussed by far-right groups were racial topics (Ottoni et al. 2018; Ben-David and Fernández 2016; Alizadeh et al. 2019; O’Callaghan et al. 2015), immigration (Ottoni et al. 2018; Ben-David and Fernández 2016) and war (Ottoni et al. 2018, being very aggressive with these topics (Mariconti et al. 2019). This conforms to expectation, as both racial content, war and immigration are topics commonly found in the far-right discourse (Panizo-LLedot et al. 2019. Interestingly, non institutional groups were more focused on a racial and anti-immigration discourse (Ben-David and Fernández 2016; Klein and Muis 2019) than the institutional far-right groups, such as political parties. Those parties were occasionally found to have a populist discourse directed against the elites (Klein and Muis 2019). The only article analysing far-left groups pinpointed that they discussed about feeling related topics more than other groups (Alizadeh et al. 2019).
- Sentiment: one of the reviewed articles (Torregrosa et al. 2020) underscored that a higher relevance in a far right community was related to a significantly higher use of negative and aggressive terminology. Similarly, the study of Figea et al. (2016) exposed that words of anger can also be useful to identify emotional concepts related to political extremist content, such as aggressiveness and concerns about other groups. Also, high negative messages were commonly forwarded against Jews, LGBT and black people (specially the first two) (Scrivens et al. 2020). Only one article (Alizadeh et al. 2019) focused on analyzing differences between far-right and far-left discourses, using a dictionary-based approach (both LIWC and Moral Foundation dictionaries). For these purpose the authors combined different NLP features to conduct a descriptive analysis from different perspectives: terms, topics and feelings. As a result, far-right was reported to use more positive words, together with terms regarding obedience to authority and pureness. By contrast, far-left resorted to more negative terms, anxiety words and terms related with justice and harm avoidance. As for the sentiment approach, this study also revealed that both groups used a general negative tone when compared to non-extremist political groups. However, from all the previously discussed outcomes, only words related to the obedience to authority yielded a significant difference.
- Semantic: Finally, the only reference to semantic analysis in political extremism related articles appears in Ottoni et al. (2018), who discerned that terms from extremist groups tend to be classified in “negative” categories using the semantic tagger from Empath. Among this category, the more relevant terms were “anger” and “violence”.

As it happened with religious extremism, far-right extremism also presented several features of the extremist discourses examined in Sect. 2.3. One of their most relevant traits is their use of specific and aggressive slang to refer to other groups. However, this is not particularly surprising, considering that some of these groups are very active on the Internet. They rely on political and historical narratives to build their discourse, also including a component of “self-victimization” therein. They also draw on hate speech and otherness as discursive resources (specially the first one, compared to religious extremism), and frequently include references to war narrative.

## NLP dataset and tools

In the analysis carried out in Sect. 4, it was noted that the data sources and the specific NLP tools in use appear frequently as relevant keywords of the works contained in the related literature corpus. This is because these elements are a fundamental part of any research work related to the study of a particular domain, in this case the extremism phenomena. The following subsections present a detailed description of both the data sources and tools used in the reviewed works, so that a complete view of the available resources is given to the audience.

### Datasets and data sources

Collecting a dataset is a key part of any NLP research process. In the case of online extremism, this step becomes specially complex, as most information represents a risk for security and/or anonymity. Therefore, it is often a hard task to find public datasets online capable of providing a solid substrate of information for modeling and/or characterization of extremism.

**Table 11:** Publicly available datasets for extremism research

Dataset	Size	Language	Source	Articles using this source
Al-Firdaws (Artificial-Intelligence-Lab 2012a)	39.715 posts—2.187 users	Arabic	Dark web forum	Chen (2008)
Montada (Artificial-Intelligence-Lab 2012d)	1.865.807 posts—52.546 users	Arabic	Dark web forum	Chen (2008)
Ansar1 (Artificial-Intelligence-Lab 2012b)	29.492 posts—382 users	English	Dark web forum	Scanlon and Gerber (2015)
How ISIS uses Twitter (Kaggle) (Fifth-Tribe 2016)	17.410 tweets—112 users	English	Twitter	Araque and Iglesias (2020); Zahra et al. (2018); Fernandez and Alani (2018); Rehman et al. (2021); Kursuncu et al. (2019); Fernandez et al. (2018); Abd-Elaal et al. (2020); Nouh et al. (2019); Gomes et al. (2017)
Automated Hate Speech Detection and the Problem of Offensive Language Davidson et al. (2017)	24.802 tweets—N/A users	English	Twitter	Johnston and Marku (2020)
Crisis Lex Dataset (not specified) (Olteanu et al. 2015)	Not specified	English	Twitter	Zahra et al. (2018)
UDI-TwitterCrawl-Aug2012 (Li et al. 2012)	50.000.000 tweets—147.909 users	English	Twitter	Agarwal and Sureka (2015)
Dataset-ATM-TwitterCrawl-Aug2013 (Li et al. 2013)	5.000.000 tweets—N/A users	English	Twitter	Agarwal and Sureka (2015)
Religious Texts Used By ISIS (Fifth-Tribe 2017)	2685 religious texts	English	Religious texts	Rehman et al. (2021); Abd-Elaal et al. (2020)
Tweets targeting ISIS (ActiveGalaXy 2016)	122.000 tweets—95.725 users	English	Twitter	Rehman et al. (2021); Abd-Elaal et al. (2020); Nouh et al. (2019)
Gawaher (Artificial-Intelligence-Lab 2012c)	372.499 posts—9.629 users	English	Dark web forum	Scrivens et al. (2015, 2018)
Turn to Islam (Artificial-Intelligence-Lab 2012e)	335.338 posts—10.858 users	English	Dark web forum	Scrivens et al. (2015, 2018)

Many of the articles included in the review use their own datasets. The reader is encouraged to contact with the authors of the different articles to ask for their data. However, in this section we deal with articles that use datasets that are either public, or can be obtained from their original source on demand. Table 11 shows a summary of the publicly available datasets used by the literature. This table contains the name of the dataset, an approximation to its size (in form of number of samples and users), the original language, the source of the data, articles using those datasets and a bibliographic reference including a link to the dataset itself.

There also exist data sources which are often used to extract textual information, but that require a preprocessing to transform them into valuable datasets for further modeling. Table 12 presents the different extremist magazines used by the literature to conduct NLP analysis. Data retrieved from these sources, however, must be curated before conducting any further analysis.

**Table 12:** Publicly available extremist data sources

Data source	Type of source	Articles using this source
Dabiq (Global-Terorrism-Research-Project 2016)	Extremist magazine	Macnair and Frank (2018); Kinney et al. (2018); Wignell et al. (2018); Bisgin et al. (2019); Araque and Iglesias (2020); Johnston and Weiss (2017); Johnston and Marku (2020); de Pablo et al. (2020); Heidarysafa et al. (2020); Skillicorn (2015)
Rumiyah (Global-Terorrism-Research-Project 2017b)	Extremist magazine	Macnair and Frank (2018); Kinney et al. (2018); Wignell et al. (2018); Araque and Iglesias (2020); Johnston and Weiss (2017); Johnston and Marku (2020); de Pablo et al. (2020); Heidarysafa et al. (2020)
Inspire (Global-Terorrism-Research-Project 2017a)	Extremist magazine	Sikos et al. (2014); Johnston and Weiss (2017); Johnston and Marku (2020); Skillicorn (2015)
Azan (Mujahid-Azhar 2016	Extremist magazine	Skillicorn (2015)

Besides the already mentioned datasets (which are part of this review), other sources might be useful for the researcher interested in obtaining more textual data related to the topics of extremism and radicalization. While these datasets are not used by the reviewed documents, and therefore fall out of this article’s scope, it is important to highlight their existence in order to assist researchers in their search for more publicly available data. As with the type of extremism of the articles in this review, they will be divided into two groups: political and religious extremism.

Concerning political extremism, a dataset of the far-right forum named Stormfront (de Gibert et al. 2018) can be found in a GitHub repository.^2^ Likewise, a dataset of alt-right users was validated by Thorburn et al. (2018), which is publicly available under request to the authors of the study. Moreover, speeches from different political parties can be accessed on the webpage of the Manifesto Project Database,^3^ with textual data corresponding to political parties with different ideologies.

Finally, related to religious extremism, the Global Terrorism Research Project (which is the source from where to download the Inspire magazine cited in Table 11) features much more content than the previously stated magazine, including a higher number of periodicals and datasets.^4^ The same holds for the AZSecure webpage, which gathers datasets from dark web jihadist forums in different languages.^5^

### Tools

While conducting a research work, it is often the case that authors inform about the tools they use for their performed experiments, along with the databases in use, for example, to create a lexicon. This section discusses on the most frequently used NLP tools when studying extremism and radicalization.

**Fig. 7:**
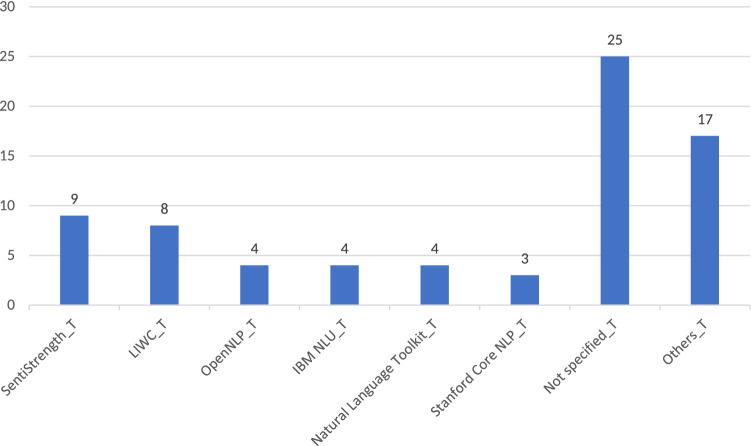
NLP tools used by the articles reviewed

Figure 7 illustrates the frequency of use of different NLP tools. Only those being used on three or more articles spawn their own category, while the rest are included under the “others” category. Also, the category “not specified” includes all those articles that do not clarify the software tools they use (Chen 2008; Alghamdi and Selamat 2012; Rowe and Saif 2016; Wei and Singh 2018; Scanlon and Gerber 2015; Hartung et al. 2017; Zahra et al. 2018; Sharif et al. 2019; Fernandez et al. 2018). As observed in this figure, the most frequently used NLP tools are:

- SentiStrength^6^: developed in 2010 (Thelwall et al. 2010), this tool was created to analyze the emotional valence (sentiment) of short texts. It uses a dictionary with sentiment related terms, from which it calculates the “strength” of the tone of different expressions. SentiStrength can report binary (positive vs negative), trinary (positive/negative/neutral) and single scale ($-4$ to $+4$) sentiment results. From the reviewed articles, it was the most commonly used tool to determine sentiment (Weir et al. 2016; Scrivens and Frank 2016; Wei et al. 2016; Saif et al. 2017; Owoeye and Weir 2019; Scrivens et al. 2015; Macnair and Frank 2018; Scrivens et al. 2020, 2018).
- Linguistic inquiry word count^7^: this tool, also known as LIWC (Pennebaker et al. 2001), was created in 2007 with the purpose of studying the language through a psychological perspective. LIWC relies on the usage of pre-established dictionaries (which can be expanded with third-party dictionaries supplied by the researcher) that are used to identify categories of words and psycho-linguistic processes underlying a text (Tausczik and Pennebaker 2010). Eight articles used it to conduct their analysis on extremism (Alizadeh et al. 2019; Hall et al. 2020; Smith et al. 2020; Sikos et al. 2014; Figea et al. 2016; Nouh et al. 2019; Torregrosa et al. 2020; Rehman et al. 2021).
- OpenNLP^8^: OpenNLP library is a ML based toolkit for the processing of natural language text,^9^ encoded in Java. It supports different NLP tasks, providing several options to analyze texts. Four reviewed articles adopted OpenNLP in their experiments (Scrivens et al. 2018, 2015; Scrivens and Frank 2016; Weir et al. 2016).
- IBM Watson natural language understanding^10^: this software, developed by IBM, includes several packages at their core, which allow conducting NLP analyzes from different perspectives (for example, open analysis versus questions and answers). This software can apply several NLP techniques to texts, such as semantic tagging, sentiment scoring or keywords and topic extraction. It was used by two articles included in the review (Ahmad et al. 2019; Wignell et al. 2018). Furthermore, the AlchemyAPI software, which was used by another two articles (Saif et al. 2017, 2016), was eventually included in the core of Watson NLU in 2015^11^.
- Natural language toolkit^12^ (NLTK): it is a NLP Python library created in 2002 (Loper and Bird 2002). It performs very similar NLP tasks than OpenNLP. Four articles used this library (Ben-David and Fernández 2016; Heidarysafa et al. 2020; Kinney et al. 2018; Klein and Muis 2019.
- Stanford Core NLP^13^: the Stanford CoreNLP is another Java based NLP tool, developed at Stanford University (Manning et al. 2014). It can perform NLP analysis in different languages, and one of its distinctive features is that it is quite easy to set up and run (Pinto et al. 2016). Three articles resorted to this NLP tool (Wei et al. 2016; Kim et al. 2017; Bisgin et al. 2019).

Even though Fig. 7 characterizes the frequency of use of the above NLP tools, other alternatives are used less frequently (namely, by less than three reviewed articles). These tools include WordNet (Bermingham et al. 2009), Stanford Maximum Entropy Part-of-speech Tagger (Bermingham et al. 2009), Vader (Wei et al. 2016; Torregrosa et al. 2020), WMatrix (Prentice et al. 2012), Gensim (Ottoni et al. 2018), iSA (Ceron et al. 2019), the Arules Package (Rekik et al. 2019), MALLET (Hall et al. 2020), the Language Detection Library for Java (Agarwal and Sureka 2015), POSIT (Weir et al. 2016; Owoeye and Weir 2018), TextRazor (Fernandez and Alani 2018), Language Model Toolkit (Mariconti et al. 2019), ConcepNet (Mariconti et al. 2019), TensorFlow Vocabulary Processor (Johnston and Marku 2020) and the Python-based tone analyzer API (Ahmad et al. 2019).

## Discussion and conclusion

This review has aimed to thoroughly explain the contributions so far of NLP to extremism research. To this end, we recall that the literature survey has been geared towards providing an informed response to several research questions posed in the introduction, regarding the different NLP issues under analysis. Throughout the whole article those issues have been analyzed, both descriptively and comparatively, based on a literature corpus included under specific targeted criteria. This last section rounds out the overview by undertaking three different purposes: the answers to the research questions mentioned previously (Sect. 8.1), a summary of future trends, challenges and directions (Sect. 8.2), and a brief conclusion with an outlook (Sect. 8.3).

### Answers to research questions

**Fig. 8:**
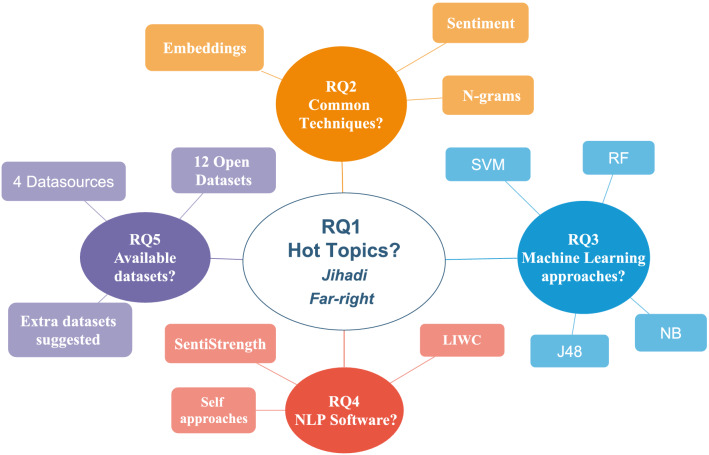
Diagram showing the main items of the replies to the posed research questions

The different research questions regarding the state of the art in NLP for extremism research were formulated in the introduction as a means to drive the methodology and analysis of the surveyed literature. Once this analysis has been completed, these research questions can now be answered by virtue of the insights drawn from the review process conducted in previous sections. Figure 8 depicts a schematic summary of the conclusions reached after the exhaustive review, highlighting the main findings for each of the research questions. Each of these answers is explained in detail below.

- RQ1. What are the current topics and contributions from NLP to extremism research? In light of the reviewed literature, with no doubt an upsurge of NLP approaches have been applied to extremism research over the last few years. Religious extremism is the most covered topic, followed by far-right extremism. Terrorism (specially Jihadist terrorism) and counter-terrorism appear to be the key motivational factors behind the interest in these topics, as detecting extremist content can help prevent radicalization processes and, thereby, avoid attacks as the ones experienced in recent years (Johansson et al. 2017). The interest in extremism detection is clearly reflected on the many mentions to ML algorithms, as their combination with NLP approaches can be useful to create classification models that allow for the accurate identification of extremist content. Finally, even though it is beyond the scope of this review, SNA also appears as an analytical approach commonly linked to the study of language in extremism research.
- RQ2. What NLP techniques are used in extremism research? Section 5 has disclosed that n-grams, TF, TF-IDF and sentiment analysis are the most commonly used techniques to study the extremist discourse. It is foreseeable that the first two approaches emerge as the most frequent in the literature, taking into account that they embody a previous step to conduct more complex analyzes, for example sentiment analysis itself. However, it should be considered that the use of neural networks models (word embeddings) is developing fast inside the community working on the study of extremist discourses, hence it should be embraced as a good starting point for researchers and newcomers interested in this topic. This is specially relevant, as authors have pointed that detecting the most commons terms used in the specific domain is not enough to understand in which meaning they are used in the text under consideration. Therefore, techniques capturing information about the context and the meaning of the terms (e.g. embedding or semantic tagging) must be considered as an important constituent part of any textual analysis in prospective contributions. This statement becomes even more substantiated taking into account that extremist texts rely on words from regular discourses, but with different objectives.
- RQ3. How have NLP techniques been applied in the field of extremism research? As stated on Sect. 6.1, 54.68% of the analyzed articles performed classification tasks using ML approaches. Again, this was expected, as the main objective of extremism research is to detect that content in advance. Among the ML algorithms in use, SVM stood out as the most commonly used one, followed by random forests, Naïve Bayes and decision trees. In terms of classification accuracy, most experiments with SVM yielded in general good performance levels. However, in the most recent research works, approaches extensively relying on different flavors of Neural Networks performed particularly well when compared to other models, and should be underlined as a promising trend for the detection of extremism based on ML classifiers. The rest of the articles (see Sect. 6.2) focused on describing the main features that differentiate between regular and extremist texts, towards uniquely defining this type of discourse. This prompted insights that could be helpful for future researchers when identifying which textual features are more useful to analyze, to detect (and eventually prevent) extremism in Social Media.
- RQ4. What NLP software tools are commonly used in extremism research? Section 7 pointed out SentiStrength as the most frequent tool to conduct NLP analysis. Specifically, this tool is used to perform sentiment scoring through the automatic tagging of words around a given token. The second one in terms of usage frequency is LIWC, a tool based on dictionaries with a psycholinguistic approach. Two further remarks should be made at this response. First, 25 articles did not report any details about the software tool utilized in their experiments. Secondly, 17 articles employed a software tool used by less of three articles. Therefore, while several NLP software tools conform a timid trend in regards to RQ4, it can be concluded that there is not a clearly dominating NLP tool in the literature for undertaking NLP analysis.
- RQ5. Which publicly available datasets or data sources have authors used to conduct NLP experiments in extremism research? Most of the articles included in the review drew on their own private datasets when conducting their experimentation. However, some of the datasets—specially those concerning religious radicalization and Twitter, forums or radical magazines—are currently publicly available to the general community. A summary of these public datasets, together with supplementary datasets suggested by the authors, have been presented in Section 7.1.

### Future trends and challenges

The research questions and their answers given above draw a general yet detailed picture of the current state of the art of NLP for extremism research. However, the literature analysis made as a requirement to inform such answers has also given rise to a manifold of insights and prospects of the future of this research area. This section outlines the future trends that the literature will follow departing from its current state, as well as the challenges that will be faced by the community will encounter and possible directions to tackle those challenges effectively.

**Fig. 9:**
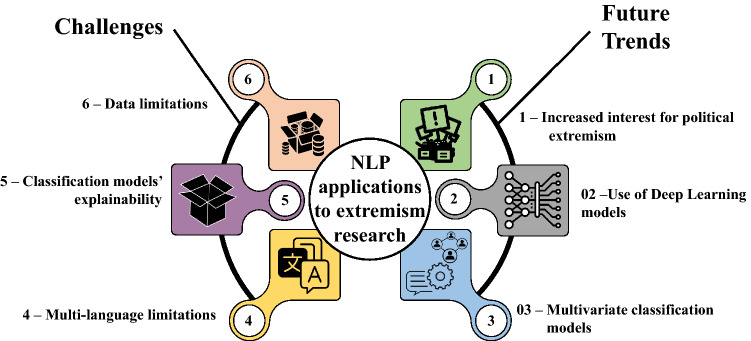
Future trends and challenges of NLP approaches applied to the extremism research area

Figure 9 shows a schematic summary of such trends and challenges. As shown in this figure, there are three main trends rooted on the research questions, and three future challenges for the NLP applications to extremism research, which are next explained in detail:

- Future trends:
  1. The relevance and global interest in political extremism will grow fast in the short term. At the time this survey is written, the Capitol assault and the shutdown of Parler (a social platform famous for being used by pro-Trump movements) have placed the political extremism under the attention of both the general public and the research community. In fact, several datasets related to online political extremism are released on a continuous basis, paving the gap towards studying this phenomenon in depth. These studies may leverage the lessons learned from the study of religious extremism. Therefore, while there are other extremist movements that will draw attention from researchers (e.g. groups like the Incels, or Involuntary Celibate, as stated on (Voroshilova and Pesterev 2021), we foresee a vibrant research activity around the detection and characterization of political extremism in future years (see, for example, Scrivens et al. (2021)).
  2. When it comes to ML for extremist prediction, neural network based techniques have showcased promising performance levels in some of the reviewed works. However, the literature approaching extremist classification with this modeling choice is relatively scarce (see also Gaikwad et al. 2021). Together with the continuous evolution of new neural architectures, the proliferation of new contributions resorting to modern neural networks is arguably a very promising trend for the future of extremism research (in similar areas, such as hate speech, a search on Google Scholar regarding deep learning shows 4 times more bibliography than with extremism). Particularly, the use of NLP approaches hinging on Deep Learning architectures (based on neural language models) also offer an effective way to overcome the lack of semantic information extracted from the texts, which is a key challenge in the study of extremist discourses (In fact, some of the upcoming research trends involve the increased use of technology based on embeddings, such as (Alatawi et al. 2021; Araque and Iglesias 2021). However, the black-box nature of these neural network models will span opportunities for explainability techniques (Arrieta et al. 2020. We envision that the explanation of these models will represent a turning point for the use of this type of approaches, issuing extended information about what these sophisticated yet opaque models observe in texts to elicit their predictions, and eventually leading to extended insights on the extremist discourse.
  3. Multivariate classification models (those fed with different types of features for discriminating among extremist and non-extremist texts) achieve in general better results in the reviewed papers. Furthermore, the general analysis carried out in Sect. 4 elucidates that some works adopt elements from social network analysis (SNA) to pursue research studies in the area of extremism. Such elements, which essentially build upon the analysis of interactions among users, could be a good complement for the study of extremist dynamics in online environments (Camacho et al. 2020). Indeed, approaches combining NLP and SNA have been investigated in other research fields, such as fake news (Zhou and Zafarani 2020), and more recently in the extremism area (Torregrosa et al. 2020), yielding good results. Also, combining these techniques with an extra layer in the analysis, such as time (e.g. Theodosiadou et al. 2021), the information obtained might be enhanced and more useful for the researcher. Therefore, it is our belief that the adoption of approaches combining techniques from different areas will take a relevant step in the analysis of extremist behaviors, not only based on textual discourses, but also on the interaction dynamics held in online social media.
- Future challenges:
  4. The presence of multiple languages in a given extremist text is a known limitation of the research area, which occurs with particular recurrence in religious extremism. This shortcoming, which is very common in this type of texts, cannot be solved just through the use of NLP techniques that may be too “simple” to overcome this problems (such as the use of n-grams instead of dictionaries). In this line, the new advances on word representation learning in NLP are enhancing the representation of the semantic information of words in word embeddings (Pilehvar and Camacho-Collados 2020), will contribute to a fine-grain processing of extremist documents. Likewise, we find in the last years a progress in the development of cross-lingual word embeddings (Søgaard et al. 2019) that represents the semantic knowledge of words from more than one language. Cross-lingual word embeddings have shown their capacity of creating a common vector space model for several languages in different tasks, as word translation and sentiment analysis (Camacho-Collados et al. 2020). Hence, the use of the last advances in cross-lingual word embeddings will alleviate addressing the challenge of multilinguality in extremist texts.
  5. Delivering interpretable explanations for the decisions issued by classification models is one of the most important challenges currently prevailing in the area, due to the psychological, criminological and sociological roots of extremism. The interest in detecting extremist content is not only justified by the detection itself, but also by the extraction of insights to gain a deeper understanding of the mind and behaviors of extremists. If this understanding is supported by explanations of the reasons why a extremist text is detected as such by a model, and if such explanations are made understandable for an audience that does not necessarily have any background in NLP, discourses can be characterized, and first signs of extremism can be identified. This can be realized by resorting to algorithmically transparent classification models (e.g. decision trees), at the cost of a potential loss of accuracy when compared to more powerful yet non-interpretable modeling counterparts, as well as by leveraging the ultimate advances in explainability and interpretability for NLP reported in recent workshops specialized in the matter (Kumar et al. 2021). Achieving a good balance between these modeling choices as per the needs of the extremism research area is a challenge that must involve multi-disciplinary views to reach a consensus on what interpretations are needed from the models to properly understand extremism in all its forms.
  6. Finally, the relative scarcity of public data sources will abide as one of the most challenging aspects to deal with in extremism research. Although massive data can be extracted from online platforms such as Twitter or web forums, the ethical concerns related to anonymity and the private nature of most data stored in such platforms prohibit researchers from sharing their datasets. This ultimately entails the creation of new datasets every time a new experiment is conducted, instead of enriching already stored datasets with new information. Therefore, creating and sharing datasets with other researchers, always respecting the ethical clauses imposed in this regard, will smooth the arrival of new researchers and teams to this field, improving the quality and quantity of the research results. We note, though, that the community is still far from this utopian stage.

### Conclusions

Currently, extremism represents a security and ideological challenge for Europe. Different kind of movements, such as jihadi terrorism and far-right groups, have changed the political and social agenda of several countries, including hot topics that are now discussed as relevant issues for those countries (Ali 2021). To confront this phenomena, it is first necessary to understand the discourse, which is a reflect of the ideology of extremist groups. Only through this understanding these movements can be prevented and counteracted.

NLP offers effective technical resources to describe these discourses, together with ways of extracting insights regarding how extremists use language compared to non-extremist groups. This review aims to achieve this objective by providing the reader with the description of:

1. The extremism itself and the concept of extremist discourse.
2. The NLP techniques used to analyze texts.
3. The different applications of these techniques.
4. Software tools and extremism datasets.

These manifold aspects of NLP for extremism research have been critically approached towards identifying future research directions, relevant trends and challenges to overcome in the study of extremist discourses (such as the need for explainable models and cross-lingual NLP techniques). Considering the insights extracted from the review, we hope that the directions, trends and challenges given in this work suggestively encourage future studies aimed at the detection and characterization of the extremist discourse in texts.

## Footnotes

1 https://www.4chan.org/.

2 https://github.com/Vicomtech/hate-speech-dataset.

3 https://manifestoproject.wzb.eu/.

4 http://gtrp.haverford.edu/resources/.

5 https://www.azsecure-data.org/dark-web-forums.html.

6 http://sentistrength.wlv.ac.uk/.

7 http://liwc.wpengine.com/.

8 https://opennlp.apache.org/.

9 https://opennlp.apache.org/docs/.

10 https://www.ibm.com/watson/natural-language-processing.

11 https://www.ibm.com/cloud/blog/announcements/bye-bye-alchemyapi.

12 https://www.nltk.org/.

13 https://stanfordnlp.github.io/CoreNLP/.
